# Toward the Design of Evidence-Based Mental Health Information Systems for People With Depression: A Systematic Literature Review and Meta-Analysis

**DOI:** 10.2196/jmir.7381

**Published:** 2017-05-31

**Authors:** Fabian Wahle, Lea Bollhalder, Tobias Kowatsch, Elgar Fleisch

**Affiliations:** ^1^ Center for Digital Health Interventions Department of Management, Technology and Economics ETH Zürich Zürich Switzerland; ^2^ Institute of Technology Management University of St Gallen St Gallen Switzerland; ^3^ Center for Digital Health Interventions Institute of Technology Management University of St Gallen St Gallen Switzerland

**Keywords:** literature review, mental health, design feature, depression, information systems

## Abstract

**Background:**

Existing research postulates a variety of components that show an impact on utilization of technology-mediated mental health information systems (MHIS) and treatment outcome. Although researchers assessed the effect of isolated design elements on the results of Web-based interventions and the associations between symptom reduction and use of components across computer and mobile phone platforms, there remains uncertainty with regard to which components of technology-mediated interventions for mental health exert the greatest therapeutic gain. Until now, no studies have presented results on the therapeutic benefit associated with specific service components of technology-mediated MHIS for depression.

**Objective:**

This systematic review aims at identifying components of technology-mediated MHIS for patients with depression. Consequently, all randomized controlled trials comparing technology-mediated treatments for depression to either waiting-list control, treatment as usual, or any other form of treatment for depression were reviewed. Updating prior reviews, this study aims to (1) assess the effectiveness of technology-supported interventions for the treatment of depression and (2) add to the debate on what components in technology-mediated MHIS for the treatment of depression should be standard of care.

**Methods:**

Systematic searches in MEDLINE, PsycINFO, and the Cochrane Library were conducted. Effect sizes for each comparison between a technology-enabled intervention and a control condition were computed using the standard mean difference (SMD). Chi-square tests were used to test for heterogeneity. Using subgroup analysis, potential sources of heterogeneity were analyzed. Publication bias was examined using visual inspection of funnel plots and Begg’s test. Qualitative data analysis was also used. In an explorative approach, a list of relevant components was extracted from the body of literature by consensus between two researchers.

**Results:**

Of 6387 studies initially identified, 45 met all inclusion criteria. Programs analyzed showed a significant trend toward reduced depressive symptoms (SMD –0.58, 95% CI –0.71 to –0.45, *P*<.001). Heterogeneity was large (I2≥76). A total of 15 components were identified.

**Conclusions:**

Technology-mediated MHIS for the treatment of depression has a consistent positive overall effect compared to controls. A total of 15 components have been identified. Further studies are needed to quantify the impact of individual components on treatment effects and to identify further components that are relevant for the design of future technology-mediated interventions for the treatment of depression and other mental disorders.

## Introduction

Over the last decade, numerous technology-mediated treatments for mental health disorders have been developed and tested in controlled trials. They form a subset of what the World Health Organization in 2005 coined “mental health information system” (MHIS). A MHIS “is a system for collecting, processing, analyzing, disseminating, and using information about a mental health service and the mental health needs of the population it serves” [1]. Although such a system does not necessarily need to rely on computerization, evidence from recent years suggests that technology-mediated MHIS holds vast opportunities in terms of much-needed scalability while ensuring treatment effectiveness. This was shown by a number of reviews and meta-analyses on computerized and Internet-delivered MHIS for mental health disorders in general [2-4] and for depression in particular [5-8].

Despite this success, it remains unclear what guides the design of MHIS and the choice of components that support existing evidence-based mental health interventions. Existing research postulates a variety of such components that show an impact on utilization of technology-mediated services and treatment outcome in general [9]. Morrison et al [10] more specifically assessed the effect of isolated components on the results of Web-based MHIS interventions and introduced that “there has been relatively little formal consideration of how differences in the design of an intervention (ie, how the content is delivered) may explain why some interventions are more effective than others.” They defined four core interactive system components that may mediate the effects of intervention design on outcomes: (1) social context and support, (2) contacts with intervention, (3) tailoring, and (4) self-management. A study by Whitton et al [11] examined the associations between symptom reduction and use of components across computer and mobile phone platforms for people with depression for one specific computerized intervention. They found that the incorporation of alert-based components, such as reminders and short motivational messages, quotes, or facts, that were sent by email or short message service (SMS) text message showed greater therapeutic gain compared with programs that did not make use of these components. At large, it was found that reminders play a decisive role in the engagement of users in mental health interventions and are a cost-effective approach for engaging users [11-13]. In addition, Landenberger and Lipsey [14] studied the relationship between specific components and the effects of computerized cognitive behavior therapy (CBT) on the recidivism of adult and juvenile offenders. Despite these findings, there remains uncertainty with regard to which components of technology-mediated interventions for mental health exert the greatest therapeutic gain across MHIS targeting people with depression. An analysis reviewing trials of technology-adaptable interventions for the treatment of depression in adults with cognitive impairments is still underway [15].

A recent preliminary literature review by Wahle and Kowatsch [16] aimed at identifying a first set of generic components for the design of MHIS for people with depression and acted as a starting point for this review in further identifying relevant components. Similar to Morrison et al [10], they hypothesized that the channel of delivery (eg, mobile phone-based, Web-based), the degree of peer support, the availability of subsidiary support, the degree of tailoring, and the existence of gamification elements likely have an impact on treatment outcome, independent from the underlying therapeutic approach [16].

This work aims at extending this list in a systematic manner and to seek evidence for the effectiveness of each of the newly identified components. By nature, MHIS represent persuasive systems. Persuasive systems may be defined as “computerized software or information systems designed to reinforce, change, or shape attitudes or behaviors or both without using coercion or deception” [17]. Therefore, we drew on Oinas-Kukkonnen and Harjumaa’s generic model of persuasive systems design [18] to identify further potentially relevant components when investigating the influence on the effectiveness of MHIS. Components the authors deemed meaningful that did not strictly follow Oinas-Kukkonnen and Harjumaa’s proposed system features were also added.

In summary, this systematic review and meta-analysis aims to add to the current body of literature by providing a systematic update in evaluating the overall effectiveness of technology-mediated treatments for depression, as well as identifying the current set of system components in use, which has not previously been conducted on a systematic review targeting depression treatments.

## Methods

An electronic search was conducted in MEDLINE, PsycINFO, and the Cochrane Library. Titles and abstracts of the identified randomized controlled trials (RCTs) were screened using predefined inclusion criteria. We independently assessed the eligibility for inclusion of all potentially relevant studies identified by the search strategy. Any disagreements were resolved by discussion among the authors. Manually screening reference lists for additional studies of relevance and tracing trials was aimed to obtain further studies possibly eligible for inclusion. Included RCTs were categorized by (1) location, (2) total number of patients randomized, (3) target condition (depression or depression comorbid with anxiety), (4) depression severity, (5) age of participants, (6) name and type of intervention, (7) type of comparator, (8) study quality, and (9) MHIS system components (see Data Extraction). A change in validated depression scores was used as the primary outcome. Data were collected from eligible trials and transferred to a data extraction table. Study quality was assessed using the widely used Jadad scale [19], additionally checking each trial for appropriate randomization, blinding of patients, as well as dropouts and withdrawals (see Assessment of Methodological Quality).

For the implementation of this systematic review, the Preferred Reporting Items for Systematic Reviews and Meta-Analyses (PRISMA) statement was used [20]. Methods of the analysis and inclusion criteria were specified in advance and documented in a protocol (can be provided on request). However, the protocol also included the evaluation of other mental disorders. Due to the large number of RCTs identified and the resulting high degree of heterogeneity, it was decided that mental disorders other than depression were not to be evaluated in this systematic review.

### Information Sources

Electronic searches were conducted in MEDLINE, PsycINFO, and the Cochrane Controlled Trials Register. Medical Subject Headings (MeSH) and relevant text word terms were used to identify relevant studies (see Search Strategy). The last search was run on September 1, 2016. Reference lists of systematic reviews and articles identified were manually checked for relevant entries.

### Search Strategy

**Figure 1:**
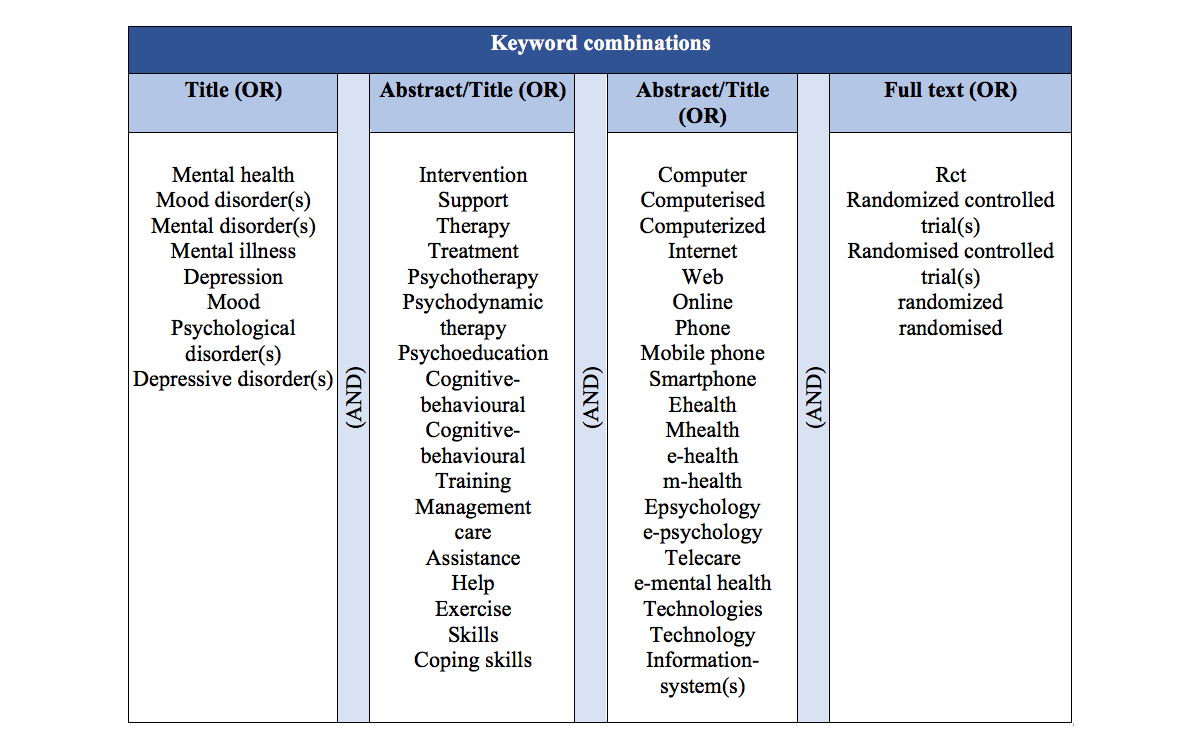
Keyword combinations used in the literature search process.

Search terms for depression were used to scan all trials registers and databases outlined previously. Additional terms for a range of delivery methods (eg, online, Internet, Web, computer, phone) and terms that specify the type of intervention (eg, cognitive behavioral, psychodynamic, interpersonal, psychoeducation) were applied. Further search terms were utilized to limit the search to studies of therapeutic interventions (eg, therapy, psychotherapy, intervention, treatment) and to RCTs. Figure 1 gives an overview of the terms used in this literature search.

As a consequence of the protocol also including the evaluation of other mental disorders, the search strategy was refined during the course of the review to limit our study to depression. A compilation of the preliminarily defined search terms is given in Figure 1.

### Study Selection

The process of study selection required an eligibility check for each article identified. Eligibility of studies was assessed by reviewing the abstracts of the references identified by the search strategy. Full texts were additionally screened when necessary. In case of doubt, any disagreements and ambiguous articles were discussed among the authors, and eligibility of studies was decided by consensus.

### Eligibility Criteria

#### Type of Study Design

Any parallel-group RCT published in English between January 2000 to September 2016 in a peer-reviewed journal was considered eligible for inclusion in this systematic analysis and synthesis.

#### Type of Participants

Studies were included if they evaluated adults or adolescents who had any of the following conditions: mild to severe depression, excluding depression co-occurring with non- *Diagnostic and Statistical Manual of Mental Disorders* (Fifth Edition) (DSM-V) [21] disorders or depression caused by environmental factors (eg, traumatic events), or depression comorbid with anxiety disorders. Studies of participants with other mental health problems (eg, schizophrenia, sleep disorders, personality disorders) were excluded to reduce the risk of heterogeneity.

#### Types of Interventions

Studies that assessed any form of technology-based intervention for depression were included in this systematic review. To assure a sufficient degree of comparability, a necessity for meta-analyses, we only included interventions in which there existed evidence for comparable outcomes for the treatment of depression. This was decided based on literature and consultation from two licensed psychotherapists. These included (1) psychotherapy (eg, cognitive behavioral therapy [22], interpersonal therapy [23], problem-solving therapy [24], supportive therapy [25]), (2) psychoeducation [26], and (3) exercise/physical activity [27] which showed results on par with a pharmacological treatment. Additional administration of drugs or procedures was allowed. The following channels for service delivery were considered eligible for inclusion: (1) offline delivery, including all interventions that did not require an Internet connectivity to provide care (eg, stand-alone computers); (2) Web-supported delivery, including interventions that made use of the Internet to deliver services (eg, using interactive websites to provide interventions or online self-help forums); and (3) mobile phone, smartphone, and tablet delivery, including all treatment programs that made use of mobile phone or tablet apps. The range of apps ranged from simple message passing to feature-rich multimedia interventions.

To meet the secondary inclusion criteria, all eligible clinical trials (according to the aforementioned eligibility criteria) were then inspected with regard to their technical feasibility. Criteria for studies being classified as technically feasible were the following: (1) to provide a methodologically structured format of care to the participants, the intervention must have adhered to a manual, protocol, or structured approach (with clearly stated processes, program structure, and objectives) and (2) treatment must not have been primarily based on face-to-face interaction, group discussion, or any other form of treatment that required personal interaction. Specific accompanying service configurations, which facilitated interaction and support with the study team and/or peer groups made possible by technology, were eligible for inclusion. In general, mental health care services, such as psychotherapeutic or behavioral interventions, were deemed suitable for the provision in a guided or nonguided format and were thus considered technically feasible. Only trials of interventions that were considered to be technically viable were included in this systematic review.

#### Types of Endpoints and Outcome Measures

In terms of types of endpoints, RCTs assessing the impact on symptoms of depression were taken into consideration. Our primary outcome measures of interest were symptoms of depression. Trials were eligible for inclusion if they have evaluated the severity of depression pre- and postintervention using one or both of these valid assessment scales: (1) the Beck Depression Inventory (BDI, BDI-1A, or BDI-II) or (2) the Patient Health Questionnaire (PHQ, PHQ-9, or PHQ-2).

The BDI is a widely used psychometric test to assess characteristic attitudes and symptoms of depression. The test consists of 21 multiple-choice self-report questions and is employed by the majority of researchers and health care professionals to measure depression severity [28]. The PHQ is a brief, self-administered assessment tool for screening and diagnosis as well as for selecting and monitoring treatment. It is part of the longer PHQ that integrates *DSM-IV* depression criteria with other leading major depressive symptoms into a concise self-report instrument [29].

#### Types of Controls

Studies were included if they compared technology-based interventions for depression to either waiting-list control (WLC), treatment as usual (TAU), or any other form of treatment for depression. The RCTs were also deemed eligible if they compared one channel of service delivery to another channel of delivery. Trials were further considered eligible if they analyzed interventions that compared different forms of subsidiary support.

#### System Components

For each identified component, we provide a rational for inclusion in the Results section. Despite some components appearing to be derived from underlying psychological theory, they were included because they were either enabled or administered by technology. For each study included in the systematic review, we determined the presence of defined system components for later analysis.

We would like to emphasize that our analysis of system components is not comprehensive and was only conducted to the degree possible based on published information in the respective literature included for the meta-review.

### Data Extraction

Data collection tables were predeveloped and subsequently refined during the process of data extraction. The following information were collected from every article.

#### Comorbidity

The occurrence of comorbidity was recorded. A difference was made between depression/depressive symptoms only and depression/depressive symptoms co-occurring with anxiety.

#### Characteristics of Intervention

The name of the therapy program and the year and location of the study were recorded. Also, information on the duration of the intervention in weeks and follow-up in months was collected. In addition, program structure and format, as well as the number of modules, were recorded. For each intervention, we further gathered any information on the aim of the intervention (inferred from the description of the intervention) and the MHIS channel(s) used (eg, online, mobile phone, computer program).

#### Characteristics of the Control Condition

Where applicable, all relevant information provided on the control condition was recorded.

#### Sample Characteristics

After applying the inclusion and exclusion criteria, we collected information on the severity of clinical depression at baseline. As a consequence of differences in the reporting of symptom severity, which was used for the inclusion/exclusion of participants, we categorized studies into one of four severity classes.

Similarly, we recorded age inclusion and exclusion criteria of all studies included in this systematic review and categorized studies into five groups (see Table 1). All data on the total size of the study population as well as on the number of participants in the treatment and control group(s) were collected.

**Table 1:** Categorization of studies according to baseline depression severity and age of participants.

Item				Rating
**Depression severity**				
	**PHQ-9**			
		0	No depression	
		1-5	Minimal depression	
		6-9	Mild depression	
		10-14	Moderate depression	
		15-19	Moderately severe	
		≥20	Severe depression	
	**BDI-II**			
		0-13	No depression	
		14-19	Minimal depression	
		20-28	Mild depression	
		≥29	Severe depression	
**Depression category**				
	0			Not reported
	1			Mild/minimal to moderate
	2			Moderately severe/severe
	3			Moderately severe to severe/major depressive episode
**Age category**				
	0			No age restrictions
	1			Adults (>16 years of age)
	2			Adolescents (14-24 years of age)
	3			Older adults (>50 years of age)
	4			Adults without older adults (18-75 years of age)

#### All Relevant Outcomes

We recorded all relevant outcomes reported on at least one of the following scores: the BDI or PHQ score.

#### Study Quality

Study quality was assessed according to Jadad et al [19] (see Assessment of Methodological Quality).

#### Service Components

For a quantified overview, the individual system components were either binary coded or, if applicable, kept in original scale.

#### Assessment of Methodological Quality

The quality of trials was examined according to the Jadad score [19] and use of intention-to-treat analysis for the available endpoints and practice of a blinded endpoint assessment. Further information can be obtained from the protocol and the quality assessment table (see Table 2).

Table2. Study quality: risk of bias in included studies (N=45). (1) Double blinded? (2) Withdrawals and dropouts reported? (3) Method of randomization reported and appropriate? (4) Method of blinding reported and appropriate? (5) Analysis “intention-to-treat”? (6) Assessment of the endpoint blinded?

**Table:** 

Study		1		2		3		4		5		6		Total score		Quality rating	
**Depression**																	
	Agyapong [30]		0.0		1.0		1.0		0.5		1.0		0.5		5.0		Good
	Andersson [31]		0.0		1.0		1.0		0.5		1.0		0.5		5.0		Good
	Andersson [32]		0.0		1.0		1.0		0.5		1.0		0.5		5.0		Good
	Berger [33]		0.0		1.0		1.0		0.0		1.0		0.5		4.5		Good
	Burton [34]		0.0		1.0		1.0		0.5		1.0		0.5		5.0		Good
	Carlbring [35]		0.0		1.0		1.0		0.0		1.0		0.5		4.5		Good
	Choi [36]		0.0		1.0		1.0		0.0		1.0		0.0		4.0		Fair
	Clarke [37]		0.0		0.5		1.0		0.0		1.0		0.0		3.5		Fair
	de Graaf [38]		0.0		1.0		0.0		0.0		1.0		0.0		3.0		Fair
	Holländare [39]		0.0		1.0		1.0		0.0		0.0		0.0		2.5		Poor
	Høifødt [40]		0.0		1.0		1.0		0.5		1.0		1.0		5.5		Good
	Johansson [41]		0.0		1.0		1.0		0.0		1.0		0.0		4.0		Fair
	Johansson [42]		0.0		1.0		1.0		0.5		0.0		0.0		3.5		Fair
	Kay-Lambkin [43]		0.0		0.5		1.0		0.5		1.0		1.0		5.0		Good
	Kessler [44]		0.0		0.5		1.0		0.5		1.0		0.0		4.0		Fair
	Kivi [45]		0.0		1.0		1.0		0.5		0.0		1.0		4.0		Fair
	Lappalainen [46]		0.0		1.0		1.0		0.0		0.0		0.0		3.0		Fair
	Lappalainen [47]		0.0		1.0		0.0		0.0		0.0		0.0		2.0		Poor
	Ly [48]		0.0		1.0		1.0		0.0		1.0		1.0		5.0		Good
	Meyer [49]		0.0		1.0		1.0		0.0		1.0		0.0		4.0		Fair
	Meyer [50]		0.0		1.0		1.0		0.0		1.0		0.0		4.0		Fair
	Morgan [51]		0.0		1.0		1.0		0.0		1.0		0.0		4.0		Fair
	Moritz [52]		0.0		1.0		0.0		0.5		1.0		0.5		4.0		Fair
	Perini [53]		0.0		1.0		1.0		0.0		1.0		0.0		4.0		Fair
	Phillips [54]		1.0		1.0		1.0		1.0		0.5		1.0		6.5		Good
	Preschl [55]		0.0		1.0		1.0		0.0		0.5		0.0		3.5		Fair
	Richards [56]		0.0		0.5		1.0		0.0		1.0		0.0		3.5		Fair
	Richards [57]		0.0		1.0		1.0		0.0		1.0		0.0		3.5		Fair
	Ruwaard [58]		0.0		1.0		1.0		0.0		1.0		0.0		4.0		Fair
	Sheeber [59]		0.0		1.0		1.0		1.0		0.0		1.0		5.0		Good
	Spek [60]		0.0		1.0		0.0		0.0		1.0		0.0		3.0		Fair
	Ström [61]		0.0		1.0		1.0		0.0		1.0		0.0		4.0		Fair
	Titov [62]		0.0		0.5		1.0		0.0		1.0		0.0		3.5		Fair
	Titov [63]		0.0		1.0		1.0		0.5		1.0		0.0		4.5		Good
	Vernmark [64]		0.0		1.0		1.0		0.5		1.0		1.0		5.5		Good
	Wagner [65]		0.0		1.0		1.0		0.0		1.0		0.0		4.0		Fair
	Watts [66]		0.0		0.5		1.0		0.0		0.0		0.0		2.5		Poor
	Agyapong [30]		0.0		1.0		1.0		0.0		1.0		0.0		4.0		Fair
**Depression and anxiety**																	
	Agyapong [30]		0.0		1.0		1.0		0.5		1.0		1.0		5.5		Good
	Andersson [31]		0.0		1.0		1.0		0.0		1.0		0.0		4.0		Fair
	Andersson [32]		0.0		1.0		1.0		0.0		1.0		0.0		4.0		Fair
	Berger [33]		0.0		1.0		1.0		0.5		1.0		0.0		4.5		Good
	Agyapong [30]		0.0		1.0		0.0		0.0		1.0		0.0		3.0		Fair
	Andersson [31]		0.0		1.0		1.0		0.0		0.0		0.0		3.0		Fair
	Andersson [32]		0.0		1.0		1.0		0.0		1.0		0.0		4.0		Fair

The quality rating was based on the total score achieved, and studies were categorized into three groups according to their quality scores: (1) good (4.5-7 points), (2) fair (3-4 points), and (3) poor (0-2.5 points). One point was given for every quality criterion met, 0.5 points for an incomplete description of the methodology used, and no points if a quality criterion was not met. As a consequence of only including RCTs in this review, it was expected that every study was described as “randomized” and thus attained at least 1 point on the quality rating score. Achieving a successful blinding in psychotherapy trials is generally considered to be very challenging, and the methods of blinding are seldom described appropriately [67]. As a consequence, we expected that only a minority of studies would reach a score higher than 5 points and consequently set the cut-off scores according to our expectations of study qualities.

#### Data Synthesis

This systematic review included a broad variety of clinical subpopulations (eg, differences in baseline severity or age) as well as treatment programs and types of comparators. Therefore, the feasibility of conducting a meta-analysis required careful consideration because the calculation of a mean treatment effect across studies could be irrelevant if studies varied significantly with regard to study populations, interventions, comparisons, or methods [68]. The protocol prespecified that if there was an adequate number of comparable studies, a random-effects meta-analysis according to the methodology of DerSimonian and Laird [69] would be conducted for the combined study groups of depression and depression comorbid with anxiety. Tables 3 and 4 provide a summarizing overview of the included studies.

**Table 3:** Summary of study characteristics, including location, sample size, study name, severity, and age of participants (N=45).

Study		Location	N	Name	Severity	Age
**Depression**						
	Agyapong [30]	Ireland	54	No name	Moderately severe to severe	Adults (≥16 years)
	Andersson [31]	Sweden	69	No name	Mild to severe	Adults (≥16 years)
	Andersson [32]	Sweden	117	No name	Mild to moderate	Adults (≥16 years)
	Berger [33]	Switzerland Germany	76	Deprexis	Mild to severe	Adults (≥16 years)
	Burton [34]	Romania Spain Scotland UK	28	Help4Mood	Mild to severe	Adults without older adults (18-75 years)
	Carlbring [35]	Sweden	80	Depressions-hjälpen	Mild to moderate	Adults (≥16 years)
	Choi [36]	Australia	63	Brighten Your Mood Program	Minimal to moderately severe	Adults (≥16 years)
	Clarke [37]	USA	160	No name	NR	Adolescents (14-24 years)
	de Graaf [38]	Netherlands	303	Colour Your Life	Mild to moderate	Adults without older adults (18-75 years)
	Holländare [39]	Sweden	84	No name	Mild	Adults (≥16 years)
	Høifødt [40]	Norway	106	MoodGYM (Norwegian Version)	Moderate to severe	Adults without older adults (18-75 years)
	Johansson [41]	Sweden	92	SUBGAP	Mild to severe	Adults (≥ 16 years)
	Johansson [42]	Sweden	121	No name	Mild to severe	Adults (≥ 16 years)
	Kay-Lambkin [43]	Australia	97	SHADE	Mild to severe	Adults (≥ 16 years)
	Kessler [44]	UK	297	No name	Mild to severe	Adults without older adults (18-75 years)
	Kivi [45]	Sweden	92	Depressions-hjälpen	Mild to moderate	Adults (≥16 years)
	Lappalainen [46]	Finland	39	Good Life Compass	Mild to severe	Adults (≥16 years)
	Lappalainen [47]	Finland	38	Good Life Compass	Mild to severe	Adults (≥16 years)
	Ly [48]	Sweden	93	No name	Mild to severe	Adults (≥16 years)
	Meyer [49]	Germany	163	Deprexis	Moderately severe to severe	Adults without older adults (18-75 years)
	Meyer [50]	Germany	396	Deprexis	NR	Adults (≥16 years)
	Morgan [51]	UK Australia Canada Ireland New Zealand USA	1736	Mood Memos	Mild to severe	Adults (≥16 years)
	Moritz [52]	Germany	210	Deprexis	NR	Adults without older adults (18-75 years)
	Perini [53]	Australia	48	Sadness	Mild to severe	Adults (≥16 years)
	Phillips [54]	UK	637	MoodGym	NR	Adults (≥16 years)
	Preschl [55]	Switzerland	40	E-mental Health Butler System	Minimal to moderate	Older adults (≥50 years)
	Richards [56]	Ireland	262	Space from Depression	Mild to moderate	Adults (≥16 years)
	Richards [57]	Ireland	101	Beating the Blues	Mild to moderate	Adolescents (14-24 years)
	Ruwaard [58]	Netherlands	54	No name	Minimal to moderate	Adults (≥16 years)
	Sheeber [59]	USA	70	Mom-Net	NR	No age restrictions
	Spek [60]	Netherlands	301	Lewinsohn’s Coping With Depression Course	Subthreshold depression	Older adults (≥50 years)
	Ström [61]	Sweden	48	No name	Mild to moderate	No age restrictions
	Titov [62]	Australia	54	Managing Your Mood Course	Mild to moderate	Older adults (≥50 years)
	Titov [63]	Australia	141	SADNESS	Mild to severe	Adults (≥16 years)
	Vernmark [64]	Sweden	88	No name	mild to moderate	Adults (≥16 years)
	Wagner [65]	Switzerland	62	No name	Minimal to severe	Adults (≥16)
	Watts [66]	Australia	52	Get Happy (Mobile app of the sadness program)	Mild to moderate	Adults (≥16 years)
**Depression and anxiety**						
	Johansson [70]	Sweden	57	No name	Moderate to severe	Adults (≥16 years)
	Mullin [71]	Australia	31	UniWellbeing Course	Minimal to moderate	Adults (≥16 years)
	Newby [72]	Australia	109	Worry and Sadness Program	Mild to severe	Adults (≥16 years)
	Proudfoot [73]	UK	167	Beating the Blues	NR	Adults without older adults (18-75 years)
	Titov [74]	Australia	290	Transdiagnostic Wellbeing Course (TD-CBT) or Disorder-Specific Mood Course (DS-CBT)	Mild to severe	Adults without older adults (18-75 years)
	Titov [75]	Australia	93	Wellbeing Course	Moderate to severe	Adults (≥16 years)
	Titov [76]	Australia	38	Wellbeing Course	Mild to severe	Adults (≥16 years)

**Table 4:** Summary of the study treatment and control groups and their relevant scores at baseline and follow-up (N=45).

Study		Treatment group^a^	Control group^a^	Baseline				Follow-up					
				Treatment		Control			Wks	Treatment		Control	
				n	Mean (SD)	n	Mean (SD)			n	Mean (SD)	n	Mean (SD)
**Depression**													
	Agyapong [30]	Supportive text messages sent by a computer + TAU	Thank you text message + TAU	26	31.58 (7.70)	28	31.99 (9.50)		13	24	8.60 (7.90)	26	16.60 (9.80)
	Andersson [31]	Guided Web-based CBT	Group CBT	33	24.00 (7.70)	36	25.30 (6.60)		9	32	13.60 (10.10)	33	17.90 (8.80)
	Andersson [32]	Web-based CBT+ Web-based discussion group	Web-based discussion group only	36	20.50 (6.70)	49	20.90 (8.50)		10	36	12.20 (6.80)	49	19.50 (8.10)
	Berger [33]	Low-intensity therapist-guided, computerized CBT	WLC	25	28.80 (8.20)	26	29.80 (8.60)		10	25	17.30 (10.20)	22	28.50 (9.40)
	Burton [34]	Help4Mood (Self-report and biometric monitoring + elements of CBT) + TAU	TAU	14	19.60 (8.10)	13	21.80 (6.80)		4	12	13.90 (8.10)	9	17.60 (6.80)
	Carlbring [35]	Web-based behavioral activation and acceptance-based treatment	WLC	40	26.32 (5.97)	40	25.13 (5.19)		8	40	16.65 (8.04)	38	23.43 (7.67)
	Choi [36]	Web-based CBT	WLC	28	25.76 (8.53)	30	20.83 (7.58)		8	23	13.48 (9.28)	28	21.27 (7.86)
	Clarke [37]	Web-based, pure self-help CBT	TAU	83	10.00 (0.80)	77	10.30 (0.80)		5	58	9.10 (0.70)	58	10.10 (0.70)
	de Graaf [38]	Computerized CBT	TAU	100	28.20 (7.70)	103	27.90 (7.50)		9	95	20.60 (10.40)	97	22.10 (10.20)
	Holländare [39]	Guided, Web-based CBT	Nonspecific support by an online therapist (email contact) + monthly rating of their depressive symptoms using the MADRS-S	42	17.00 (11.50)	42	17.70 (11.50)		10	38	9.30 (12.00)	39	13.40 (11.90)
	Høifødt [40]	Guided, Web-based CBT (TAU)	WLC (TAU)	52	21.13 (6.85)	54	22.27 (6.74)		7	52	14.20 (8.15)	54	18.63 (8.64)
	Johansson [41]	Web-based psychodynamic psychotherapy + online therapist contact	Web-based structured support intervention (psychoeducation and scheduled weekly contacts online)	46	26.54 (5.80)	46	26.33 (6.70)		10	42	11.48 (7.80)	46	20.22 (7.80)
	Johansson [42]	Tailored Web-based CBT	Web-based discussion group with weekly discussion themes related to depression and the treatment of depression	36	26.44 (7.60)	42	26.24 (7.90)		10	36	13.78 (9.40)	39	21.67 (9.50)
	Kay-Lambkin [43]	Computerized CBT therapy and motivational interviewing by a computer program	No further treatment	23	28.57 (9.89)	21	32.86 (9.59)		13	23	17.09 (12.14)	21	22.95 (10.46)
	Kessler [44]	Web-based CBT + TAU	WLC (TAU)	149	32.80 (8.30)	148	33.50 (9.30)		17	113	14.50 (11.20)	97	22.00 (13.50)
	Kivi [45]	Web-based CBT	TAU	45	25.50 (7.87)	47	26.09 (9.39)		13	30	13.23 (10.94)	35	14.46 (9.88)
	Lappalainen [46]	Guided Web-based acceptance and commitment therapy without face-to-face contact	WLC	19	22.11 (7.79)	20	20.65 (6.80)		7	18	13.34 (6.75)	20	17.85 (7.34)
	Lappalainen [47]	Guided Web-based acceptance and commitment therapy	Face-to-face Acceptance and Commitment therapy (ACT)	19	20.79 (9.34)	19	23.11 (6.38)		6	19	10.26 (8.20)	18	9.17 (5.24)
	Ly [48]	Blended treatment (4 face-to-face sessions + a smartphone application used between sessions)	Full behavioral activation	46	28.96 (8.07)	47	27.32 (7.89)		9	44	15.17 (11.51)	46	13.43 (11.27)
	Meyer [49]	Web-based CBT + TAU	WLC (TAU)	78	16.62 (3.44)	85	17.20 (3.86)		9	60	10.08 (6.37)	73	13.64 (6.14)
	Meyer [50]	Web-based CBT + TAU	WLC (TAU)	320	26.72 (9.86)	76	27.11 (8.98)		9	159	19.87 (11.85)	57	27.15 (10.01)
	Morgan [51]	Emails promoting the use of self-help strategies	Emails containing depression information	862	16.40 (5.98)	874	16.90 (5.76)		6	273	10.80 (6.84)	301	11.50 (6.72)
	Moritz [52]	Web-based CBT	WLC	105	28.81 (11.11)	105	30.02 (10.18)		8	80	20.51 (12.22)	90	25.67 (11.65)
	Perini [53]	Guided Web-based CBT	WLC	27	27.30 (7.30)	18	27.24 (6.18)		8	27	17.30 (9.86)	17	23.33 (9.29)
	Phillips [54]	Computer-based CBT	Attention control (5 websites with general information about mental health)	311	14.60 (5.40)	318	14.60 (5.60)		6	164	9.90 (6.10)	176	10.20 (6.00)
	Preschl [55]	Face-to-face life-review therapy including computer supplements	WLC	20	19.00 (6.60)	16	16.50 (5.60)		8	20	10.00 (6.30)	16	15.10 (7.80)
	Richards [56]	Guided Web-based CBT	WLC	96	20.90 (3.83)	92	20.84 (4.17)		8	96	15.67 (7.68)	92	20.43 (6.97)
	Richards [57]	Unguided Web-based CBT	Therapist-assisted email CBT	43	21.72 (5.30)	37	22.70 (4.70)		8	21	12.81 (6.90)	25	11.52 (4.90)
	Ruwaard [58]	Guided Web-based CBT	WLC	36	19.70 (5.50)	18	21.30 (5.30)		11	36	9.80 (6.50)	18	15.60 (7.60)
	Sheeber [59]	Guided Web-based CBT	WLC (TAU)	35	26.20 (9.80)	35	25.40 (9.00)		14	34	13.40 (10.40)	35	22.50 (11.00)
	Spek [60]	Unguided Web-based CBT	Group face-to-face CBT	102	19.17 (7.21)	99	17.89 (9.95)		10	67	11.97 (8.05)	56	11.43 (9.41)
	Ström [61]	Therapist-guided Web-based physical activity (guided self-help program)	WLC	24	26.92 (9.30)	24	28.25 (7.08)		9	24	17.88 (11.30)	24	24.04 (6.86)
	Titov [62]	Guided Web-based CBT	WLC	27	11.04 (5.62)	25	12.04 (5.42)		8	23	3.96 (2.48)	22	12.68 (5.48)
	Titov [63]	Clinician-guided Web-based CBT	WLC	41	27.15 (9.96)	40	26.33 (10.46)		8	41	15.29 (9.81) 7.59 (4.04)	40	26.15 (10.14)
	Vernmark [64]	Web-based CBT, email supported	WLC	30	22.20 (5.30)	29	21.80 (6.60)		8	29	10.30 (5.20)	29	16.60 (7.90)
	Wagner [65]	Guided Web-based CBT	Face-to-face CBT	32	22.96 (6.07)	30	23.41 (7.63)		8	25	12.41 (10.03)	28	12.33 (8.77)
	Watts [66]	Smartphone-based CBT	Computer-based CBT	15	33.46 (2.95)	20	30.90 (2.55)		8	1010	12.53 (3.26)	15	13.68 (2.79)
											
**Depression and anxiety**													
	Johansson [70]	Web-based psychodynamic, guided self-help treatment based on affect-phobia therapy (APT)	WLC	28	15.32 (3.30)	29	15.07 (4.40)		10	28	5.89 (2.80)	29	10.59 (6.40)
	Mullin [71]	Web-based CBT (total sample: N=30); clinical subsample (PHQ-9 ≥10)	WLC	20	14.10 (3.62)	11	14.63 (3.35)		6	20	8.33 (4.86)	11	13.37 (7.42)
	Newby [72]	Guided Web-based CBT (Worry and Sadness Program)	WLC	46	21.24 (6.98)	54	22.41 (9.17)		10	43	10.48 (8.30)	53	21.24 (10.56)
	Proudfoot [73]	Computerized CBT	TAU	53	25.38 (11.05)	53	24.08 (9.78)		8	47	12.04 (10.45)	50	18.36 (12.65)
	Titov [74]	Clinician-guided Web-based CBT	Self-guided Web-based CBT	112	15.07 (3.57)	105	15.23 (3.85)		8	112	7.36 (5.04)	105	8.44 (5.14)
	Titov [75]	Transdiagnostic self-guided Web-based CBT with automated email	self-guided Web-based CBT without automated email	47	14.64 (3.34)	46	14.39 (3.33)		8	47	7.58 (4.60)	46	10.57 (6.16)
	Titov [76]	Web-based CBT with email and phone support	WLC	18	14.39 (4.27)	20	13.35 (6.25)		10	18	7.67 (5.97)	20	12.15 (4.93)

^a^ BDI: Beck Depression Index; CBT: cognitive behavioral therapy; PHQ: Patient Health Questionnaire; TAU: treatment as usual; WLC: waiting-list control.

### Statistical Analyses

Each study was summarized in detail in the predeveloped data extraction table. The primary outcome of the BDI and PHQ was assessed as a continuous measure of effect in an additional table. Because moderate to substantial heterogeneity among the interventions was expected, mean effect sizes were calculated using a random-effects meta-analysis according to DerSimonian and Laird [69]. Review Manager 5 (RevMan 5) was used to conduct this systematic review [77]. In general, a *P* value of <.05 was considered statistically significant.

#### Calculation of Effect Sizes: Changes in Primary Outcome Measures Between Pre- and Posttreatment

For every technology-based intervention, to assess the within-group effect (uncontrolled effect size) of treatments, we calculated the standard mean difference (SMD) as effect size referring to the difference between baseline and postintervention, divided by the pooled standard deviation of each primary outcome measure, and the 95% confidence intervals around the effect sizes. According to the methodology described in Hedges [78], effect sizes were also adjusted to address small sample sizes. As a consequence of the interdependency of baseline and posttreatment values, the correlation between time points was required. However, because the majority of included studies did not provide the correlation between these values, a conservative value of .50 was used as suggested by Balk et al [79].

#### Calculation of Effect Sizes: Technology-Based Interventions Versus Control Conditions

We calculated the effect size (SMD or Hedges’ *g* [78]) for each comparison between a technology-enabled intervention and a control condition. It indicates the difference between the two study groups at posttest (standardized mean difference) and the 95% confidence intervals around the effect sizes. Effect sizes resulted from the subtraction of the mean score of the intervention group at posttreatment from the mean score of the comparator group and dividing the result by the pooled standard deviation of the two groups. Values of 0.8 refer to large, 0.5 to moderate, and 0.2 to small effects [80]. To address small sample sizes, we also adjusted effect sizes according to the procedures described by Hedges [78]. Only those instruments that measured depressive symptoms were used in the calculation of effect sizes.

In cases in which more than one depression measure was provided, the BDI was preferred over the PHQ. If studies only assessed the PHQ score, the PHQ was used for calculations. In this analysis, only the effect sizes referring to the differences between the two study groups at posttreatment were used. Because the follow-up period varied considerably between studies, we decided not to examine the differential effects at these time points.

#### Assessment of Heterogeneity

As a consequence of the anticipated moderate-to-high level of diversity between study populations and interventions eligible for this systematic review, the Breslow-Day test was used to test for heterogeneity [81]. To complement the common chi-square test for heterogeneity, the I^2^ statistic proposed by Higgins et al [82] was used. Inconsistency (termed I^2^) was calculated by the formula: I^2^=max 0, 100%*(Q-df)/Q, where *Q* is the heterogeneity statistic and *df* its degrees of freedom. Because I^2^ is not inherently dependent on the number of studies, this characteristic is of advantage in assessing the percentage of total variation across studies due to heterogeneity. An I^2^ value greater than 50% was considered as strong inconsistency [83].

#### Subgroup and Sensitivity Analyses

Because a high degree of heterogeneity was to be expected, we tried to mitigate this issue by subgroup analyses. We tested prespecified hypotheses to assess the robustness of the findings and to explore sources of heterogeneity (relationships between study characteristics and intervention effects). The following hypotheses were proposed. It was assumed that the treatment effect was influenced by (1) duration of treatment, (2) severity of depression (baseline score), (3) age of participants, (4) methodological quality of studies, (5) type of control (eg, TAU, WLC), (6) inclusion of face-to-face therapist sessions, and (7) utilization of CBT techniques.

#### Assessment of Publication Bias

The data collection was based on the description of interventions in published literature. Thus, grey literature assessing the effectiveness of technology-based interventions was not taken into account. The potential presence of publication bias likely had a significant impact on the results of this study, not only with respect to differences in usage of online interventions in clinical settings, but also in more real-world settings [84]. To improve and standardize the description of technology-based interventions, it is suggested that future studies apply frameworks such as the Consolidated Standards of Reporting Trials (CONSORT) statement for eHealth [85], a protocol for systematic reviews [86] and guidelines for reporting online intervention research [73]. Language bias might be an issue in this review because only RCTs published in the English language were included. These limiting factors should be kept in mind when interpreting the findings of the current work. In order to identify cases of possible publication bias, a funnel plot was drawn for the main analysis [87]. Nonpublication of small trials would result in asymmetry of the plot. In addition, the funnel plot was evaluated for asymmetrical distributions. To confirm the visual interpretation, which can be subjective, the Begg and Mazumdar [88] adjusted rank correlation test for publication bias was used.

## Results

In this section, the findings of the different analyses that were carried out in this review are reported. Characteristics of studies are presented in tabular form.

### Study Selection

**Figure 2:**
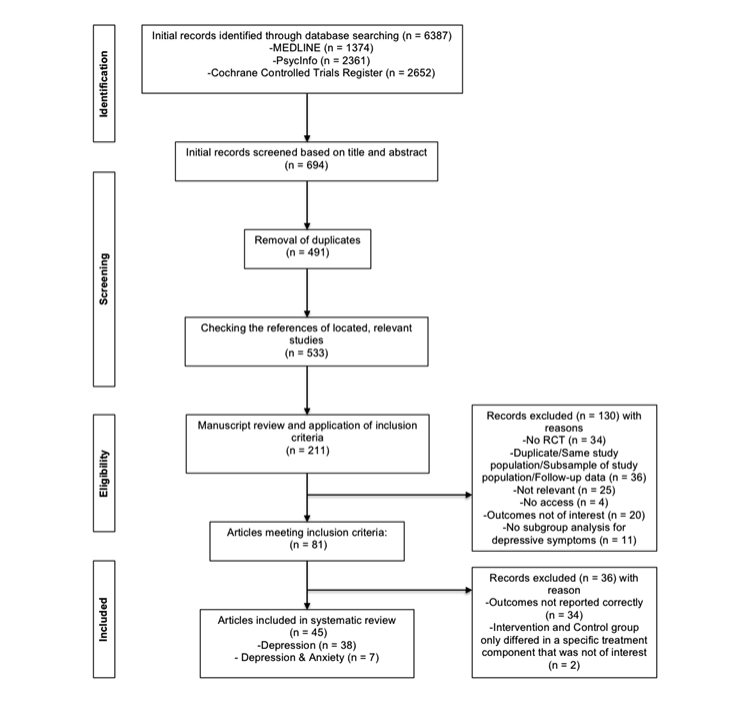
Flow diagram of study selection.

The searches in MEDLINE, PsycInfo, and the Cochrane Controlled Trials Register identified a total of 6387 citations (articles and abstracts) published after 2000. After the adjustment for duplicates and the exclusion of noneligible trials based on titles and abstracts, 491 studies remained. Forty-two additional possibly eligible trials were identified by checking the reference lists of relevant articles already identified. A more detailed review of the full text of the remaining citations led to the detection and exclusion of 130 publications. Thirty-four trials were excluded because of the lack of appropriate reporting of outcomes. It was decided by consensus to exclude two additional studies that included an active control group that only differed from the study group in the use of a program component that was not relevant to this review. In total, 45 RCTs were included. Of these, seven trials analyzed patients with depressive symptoms comorbid with anxiety. Figure 2 summarizes the study selection process.

### Characteristics of Studies and Risk of Bias Within Studies

Tables 3 and 4 provide information on the setting and outcomes of the 45 trials finally included in the analysis. These RCTs contributed a total of 7326 randomized and 4519 analyzable patients. The majority of trials studied CBT in adult patients with mild-to-moderate depressive symptoms.

The risk of bias at the level of the individual trials is addressed in Table 2 by reporting the modified Jadad score [19] and whether analysis was performed according to the intention-to-treat principle (see Assessment of Methodological Quality). None of the trials were described as “double-blind RCT” and we also noted a relatively high risk of bias due to the insufficient blinding of participants. Typically, the method of blinding was described insufficiently. This, however, is not uncommon for psychotherapy trials [67]. In general, achieving a successful blinding in psychotherapy trials is regarded to be more demanding than in pharmacological trials [89]. Study participants can easily identify the discrepancies in the contents of the treatment and control arms, and it is probably not very likely to successfully blind the participants or the therapists. Thus, the methods used to achieve proper blinding are rarely reported in psychotherapy trials [67]. The risk of bias introduced by selective reporting was small because all outcomes of interest were adequately described in the vast majority of the included RCTs.

### System Components

In total, a set of 15 system components was identified based on occurrence in reviewed literature. These were either defined, hypotheses-driven, or derived from Oinas-Kukkonnen’s model of persuasive systems design [18]. For each study included in the systematic review, we determined the presence of defined system components for later analysis. In the following, we present an overview of identified components, together with the underlying inclusion rationale.

#### Channel of Delivery

Technology-mediated MHIS can be administered using a range of available technologies. Although early interventions were based on offline programs, computerized programs and Internet-delivered Web interventions have become more popular in recent years [16]. Following the latest development, the mobile phone as a channel of delivery is getting more and more attention. This seems reasonable because almost half of the world’s population has a mobile phone subscription, and it is expected that by 2020 the global penetration rate will reach approximately 60% [90]. In addition, it is suggested that differences in access to mobile technologies are diminishing at least for nonrural populations, thus offering an opportunity to reach underserved and marginalized populations [91]. The high global penetration and the rapid growth of mobile phone apps provide the opportunity to reach an increasing number of people who are in need of treatment for mental disorders [92].

#### Tailoring (Personalization)

Oinas-Kukkonen argued that “information provided by the system will be more persuasive if it is tailored to the potential needs, interests, personality, usage context, or other factors relevant to a user group” and that “a system that offers personalized content or services has a greater capability for persuasion” [18]. This was confirmed in the context of health behavior change by a systematic review [93] and presents a promising component for the treatment of depression in MHIS.

#### Supportive Text Messages (Tunneling/Praise)

Research by Agyapong et al [30] targeting the support for people with depression and comorbid alcohol use disorder presented promising results in the deployment of supportive text messages. This followed the Oinas-Kukkonen concept of tunneling [18] (ie, “using the system to guide users through a process or experience provides opportunities to persuade along the way”) and the concept of praise, which is said to make users more open to persuasion [17].

#### Peer Support

Although no consensus with respect to effectiveness of online peer support was reached yet [94,95], anonymous online support groups and discussion forums might help users to overcome the feeling of being stigmatized by connecting patients with others. A further advantage of these social support components is that time and location are no longer obstacles for active participation [96]. Peer support follows the Oinas-Kukkonens [18] concept of social learning, social comparison, and social facilitation [96].

#### Case-Enhanced Learning

This form of learning uses educational stories that identify a problem and a solution with an example (ie, a case) the participant can potentially identify with [75]. These can be implemented via, for example, video vignettes of case-study patients.

#### Reminders

Stemming from Oinas-Kikkonens concept of reminders, Whitton et al [11] found that reminders play a decisive role in the engagement of users in mental health interventions and are a cost-effective approach for engaging users [11-13]. Furthermore, it is suggested that reminders not only enhance user engagement but also improve adherence [97-99] and counteract the high rates of nonusage attrition common to many online-based interventions [97,100].

#### Downloadable Material

Because a participant’s preferred medium for reading might be paper [101], an option to download and print out summaries, lessons, or homework might influence treatment efficacy by providing a higher level of comfort.

#### Workbook/Homework Assignments

Homework assignments, as commonly used in standard care [102], are an important construct in CBT. In a recent study, LeBeau et al [103] concluded that “improvement of homework compliance has the potential to be a highly practical and effective way to improve clinical outcomes in CBT.” Therefore, implemented in an appealing interactive way, this might represent an important component in MHIS.

#### Symptom Tracking

Tracking symptoms, either objectively using sensors [104] or by means of self-reports might be beneficial for the user following Oinas-Kikkonens self-monitoring concept [18]. It describes a “system that helps track one’s own performance or status supports in achieving goals” [18].

#### Online Diary

Diaries form a way of self-monitoring and self-reflection and are frequently used in classical forms of CBT [105].

#### Summaries

We assessed whether included studies made use of summaries of content (eg, module or progress summaries), which represent another dimension drawing on the concept of self-monitoring and self-reflection [17].

#### Audio/Voiceover

A recent experimental study found that audience feedback is a valuable tool to enhance users’ perceptions of health-related YouTube clips [106], which highlights the power of the participatory nature of the Web to increase the efficacy of Internet-based health interventions.

#### Illustrative Content/Video

Illustrative content in the form of graphics, photos, illustrations, comics, or video clips might increase the appeal of interactivity and visual attractiveness of Internet-based programs [107].

#### Gamification

The use of game-like strategies has demonstrated to produce positive outcomes in previous studies of technology-based health interventions [108,109]. Gamification of health apps might provide the option to set up goals and rules for personal health behavior and to track patient’s actual behavior against these rules and goals [108]. The utilization of rewards in the context of health intervention might be promising because it was found that rewards are able to stimulate positive thinking in users and are thus a powerful tool to drive long-term participation [110].

#### Animations/Virtual Assistant

Virtual agents or avatars could be used for persuasive purposes and to support self-management among patients [111]. A growing body of literature examines the relationship of virtual agents and their user, potentially holding vast opportunities for persuasive system design [112-114].

### System Component Setting

Table 5 contains detailed information on the settings and system component configuration of the interventions.

Approximately 45% (20/45) of all included studies reported using email reminders and 10 of 45 studies (22%) reported providing SMS text message reminders, mostly as an alternative to reminders sent by email. In the included trials, reminders were typically intended to increase motivation and adherence to therapeutic interventions. As explained earlier, reminders play a decisive role in the engagement of users in mental health interventions. Most of the RCTs made use of the Internet for delivering mental health interventions for depression or depression comorbid with anxiety. The majority of RCTs included in this systematic review (91%, 41/45) did not make use of mobile phones or tablets. Typically, the interventions required interaction with the system, and many also included interaction with a therapist (face-to-face or online) and/or peers on the Web. In all, 80% (36/45) of included programs were based on CBT or used elements of CBT. Tunneling, which refers to the stepwise delivery of content, is typically found in technology-based interventions for depression [18], and was also used in the majority of studies (87%, 39/45) included in this work. Twenty-seven of 45 included studies used tailored content, tailored feedback, and/or tailored reminders.

Only a small number of included studies made use of self-monitoring components, such as symptom tracking and tracking reminders, yet they are seen as key features of psychotherapy in particular [40,96], and in behavior-change interventions in general [12,115-118].

Although social support is widely recognized as an important feature in behavior change [119,120], in this analysis, only seven studies (16%) used peer support. In the included studies, social support consisted of the use of Web-based discussion boards (asynchronous social support), which was intended to provide the ability to connect with other patients of the same intervention.

Regarding visual attractiveness, 44% of all included studies analyzed an intervention that included visually appealing content, such as graphs, illustrations, comics, or photos, which often serve a motivational purpose. Only 11 of 45 studies (24%) described the utilization of audio and/or voiceovers. Video footage, often containing case-enhanced learning, was also found in 11 RCTs (24%).

**Figure 3:**
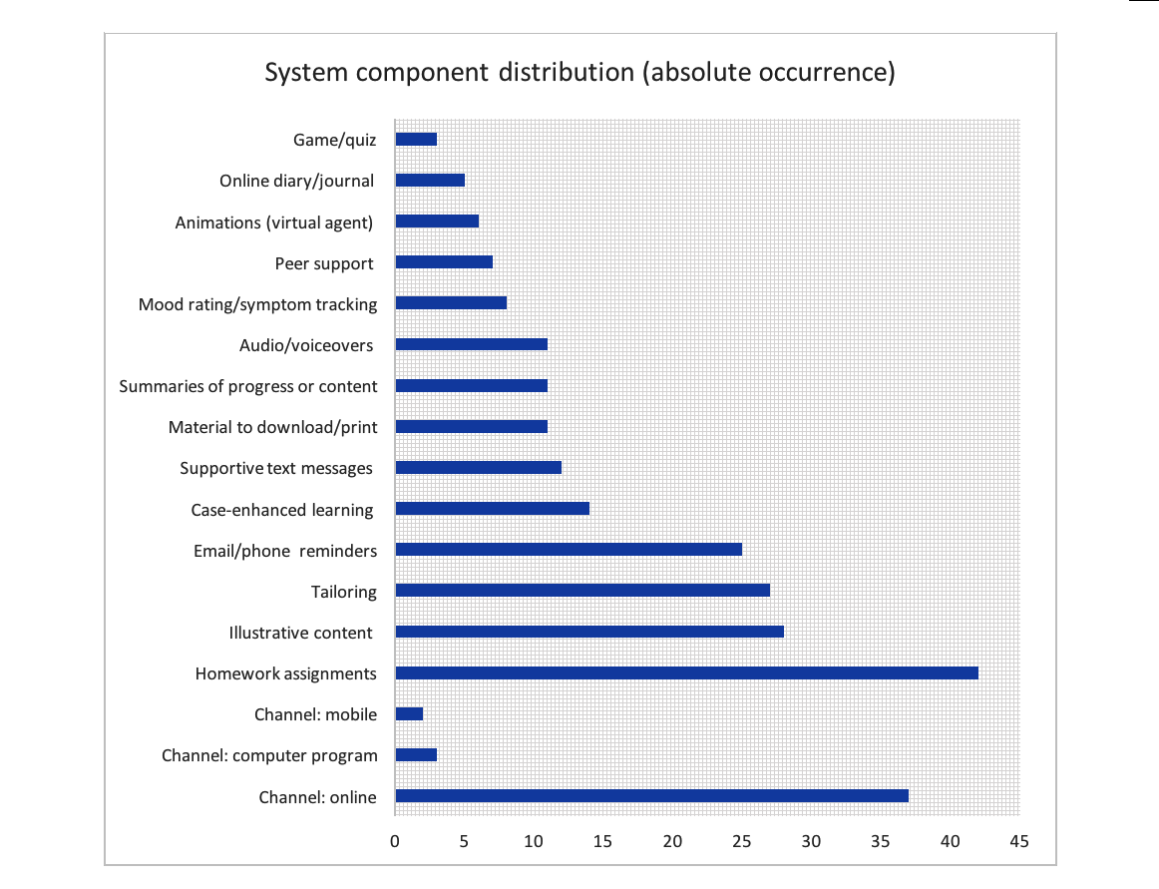
System component distribution of included studies (N=45).

In addition, this analysis showed that game elements were only used in three RCTs and, if gaming was included, it was in the form of knowledge quizzes. See Figure 3 for a quantitative overview of components among included studies.

**Table 5:** MHIS system component configuration of included studies for health information system (HIS) channel and tailoring and (1) email/phone reminders, (2) supportive text messages, (3) peer support, (4) summaries of progress or content, (5) case-enhanced learning, (6) material to download/print, (7) homework assignments, (8) mood rating / symptom tracking, (9) online diary/journal, (10) audio/voiceovers, (11) illustrative content, (12) games/quizzes, and (13) animations (virtual agent).^a^

Study		HIS channel	Tailoring	1	2	3	4	5	6	7	8	9	10	11	12	13
**Depression**																
	Agyapong [30]	Mobile phone	0/NR	1	1	0	0	0	0	1	0	0	0	0	0	0
	Andersson [31]	Online	0/NR	0	0	0	0	0	0	1	0	0	0	0	0	0
	Andersson [32]	Online	Partly	1	0	1	0	0	1	1	0	0	0	0	1	0
	Berger [33]	Online	1	1	0	0	0	0	1	1	0	0	0	1	0	0
	Burton [34]	Online	1	0	0	0	1	0	0	0	1	0	1	0	0	1
	Carlbring [35]	Email	1	0	0	0	0	0	0	1	0	0	1	1	0	1
	Choi [36]	Online	0/NR	1	0	0	1	1	0	1	0	0	0	1	0	0
	Clarke [37]	Online	1	1	0	1	0	0	0	1	1	1	0	1	0	0
	de Graaf [38]	Online	0/NR	0	1	0	0	0	0	1	1	1	0	1	0	0
	Holländare [39]	Online	0/NR	1	0	0	0	0	0	1	1	0	0	1	0	0
	Høifødt [40]	Online	Feedback/reminders	1	0	0	0	0	0	1	0	0	0	1	0	0
	Johansson [41]	Online	Feedback/reminders	1	1	0	0	0	0	1	0	0	0	0	0	0
	Johansson [42]	Online	1	1	1	1	0	0	1	1	0	0	0	0	0	0
	Kay-Lambkin [43]	Computer program	0/NR	0	0	0	0	0	0	1	0	0	1	1	0	0
	Kessler [44]	Online	1	0	1	0	0	0	0	0	0	0	0	0	0	0
	Kivi [45]	Online	Feedback/reminders	0	0	0	0	0	1	1	0	1	1	1	0	0
	Lappalainen [46]	Online	Feedback/reminders	1	0	0	0	0	0	1	0	1	1	1	0	0
	Lappalainen [47]	Online	1	1	0	0	0	0	0	1	1	0	1	0	0	0
	Ly [48]	Mobile phone	1	1	1	0	0	1	0	1	0	0	0	1	0	0
	Meyer [49]	Online	1	1	1	0	0	0	1	1	1	0	1	1	0	0
	Meyer [50]	Online	1	0	0	0	0	1	0	1	0	0	0	1	0	1
	Morgan [51]	Email	0/NR	1	1	0	0	0	0	0	0	0	0	0	0	0
	Moritz [52]	Online	1	0	0	0	0	1	0	1	0	0	0	1	0	1
	Perini [53]	Online	0/NR	0	0	1	1	1	1	1	0	0	0	1	0	0
	Phillips [54]	Online	Feedback/reminders	1	0	0	0	0	0	1	0	0	0	1	0	0
	Preschl [55]	Computer program	1	0	0	0	0	0	1	1	0	0	1	1	0	0
	Richards [56]	Online	1	1	0	0	1	1	0	1	0	0	0	1	1	0
	Richards [57]	Online	Feedback /reminders	0	0	0	0	0	0	1	0	1	1	1	0	1
	Ruwaard [58]	Online	1	0	1	0	0	0	0	1	1	0	0	0	0	0
	Sheeber [59]	Online	Feedback/reminders	0	0	1	0	0	0	1	1	0	1	0	0	0
	Spek [60]	Online	0/NR	0	0	0	0	0	0	1	0	0	0	1	0	0
	Ström [61]	Online	1	0	1	0	0	0	0	1	0	0	0	0	0	0
	Titov [62]	Online	0/NR	1	0	0	1	1	0	1	0	0	0	0	0	0
	Titov [63]	Online	0/NR	1	0	1	1	1	1	1	0	0	0	1	0	0
	Vernmark [64]	Online	1	1	0	0	0	0	1	1	0	0	0	0	1	0
	Wagner [65]	Online	Feedback/reminders	0	0	0	0	0	0	1	0	0	0	0	0	0
	Watts [66]	Mobile phone vs PC	0/NR	0	0	0	0	1	0	1	0	0	0	1	0	0
	Agyapong [30]	Online	0/NR	1	0	0	0	0	0	1	0	0	0	0	0	0
**Depression and anxiety**																
	Johansson [70]	Online	Feedback/reminders	0	0	0	0	0	0	1	0	0	0	1	0	0
	Mullin [71]	Online	0/NR	1	0	0	1	1	0	1	0	0	0	1	0	0
	Newby [72]	Online	0/NR	1	0	0	1	1	1	1	0	0	0	0	0	0
	Proudfoot [73]	Computer program	1	NR	0	0	1	0	1	1	0	0	1	1	0	1
	Titov [74]	Online	0/NR	1	1	0	1	1	0	1	0	0	0	1	0	0
	Titov [75]	Online	0/NR	1	0	0	0	1	0	1	0	0	0	1	0	0
	Titov [76]	Online	0/NR	1	1	1	1	1	0	1	0	0	0	1	0	0

^a^ Rating system is 1=component present and 0=component not present. CG: control group; NR: not reported; TG: treatment group

### Synthesis of Results: Impact on Symptoms of Depression

**Figure 4:**
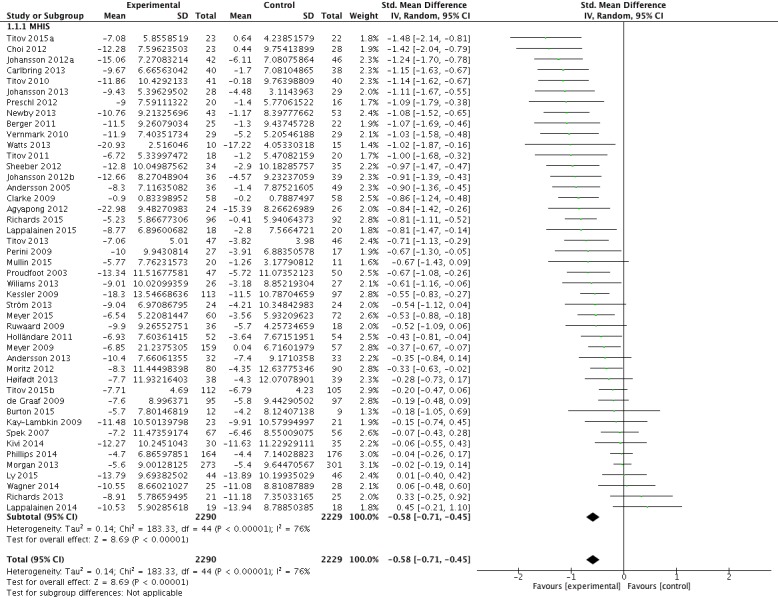
Effect of technology-based interventions on symptoms of depression in included studies (N=45).

Data from 45 trials (4519 patients) that reported on BDI or PHQ scores before and after the treatment were combined to estimate the overall effect of technology-based interventions on depressive symptomatology. Technology-supported treatments for depression showed a trend toward reduced depressive symptoms (SMD=–0.58, 95% CI –0.71 to –0.45; *P*<.001) (Figure 4). The chi-square test for heterogeneity (χ^2^_44_=183.3), across all patient types, was significant (*P*<.001) and total variation across trials due to heterogeneity (I^2^) was 76%, a strong indication for inconsistency across included RCTs.

### Risk of Bias Across Studies

**Figure 5:**
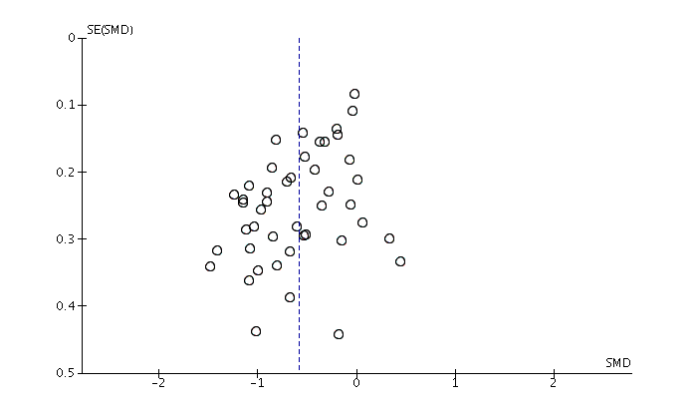
Risk of bias across all included studies (N=45).

The funnel plot drawn for the main analysis on the effectiveness of technology-delivered interventions for depression showed an asymmetry, which is evidence of missing studies suggesting publication bias (Figure 5). Additional testing utilizing the Begg and Mazumdar rank correlation method [88] confirmed that there is, in fact, evidence of publication bias (*P*=.03).

As a consequence of this finding, we also explored publication bias in the 22 RCTs that used a WLC group. The funnel plot of this subgroup of included trials showed no evidence of bias, which was confirmed by the Begg test (*P*=.08).

### Results of Subgroup Analyses

Subgroup analyses by study quality, treatment duration, provision of face-to-face contact, utilization of CBT techniques, severity of baseline depression, age of participants, and type of control (eg, TAU or WLC) were prespecified and served to test our hypotheses (see Subgroup and Sensitivity Analyses *).* The individual forest plots per subgroup analysis can be found in Multimedia Appendixes 1-7.

### Study Quality

Differences in study findings could also be explained by biased results due to differences in quality of individual studies. Thus, a subgroup analysis based on the methodological quality of included trials was performed. It could be shown that the effect of technology-based interventions on symptoms of depression is consistent in trials of higher (quality score >3.5) and lower quality (quality score ≤3.5). In high-quality trials, technology-based interventions were associated with an SMD of –0.60 (95% CI –0.76 to –0.45, *P*<.001; I^2^=77%) and a SMD of –0.53 (95% CI –0.77 to –0.29, *P*<.001; I^2^=76%) in low-quality studies. It seems that methodological quality of trials only showed a small impact on effect sizes, but overall had a moderate effect (χ^2^_1_=0.3, *P*=.60; I^2^=0%).

### Treatment Duration

As statistical heterogeneity was found and to exclude the possibility of heterogeneity due to differences in the duration of the interventions, trials of different durations were compared to one another. Regarding depressive symptomatology, technology-based interventions showed to be effective, irrespective of treatment duration. In treatments with a duration of 10 weeks or less, the intervention was associated with an SMD of –0.60 (95% CI –0.76 to –0.44, *P*<.001; I^2^=79%). Similarly, treatments with a duration of longer than 10 weeks resulted in an SMD of –0.52 (95% CI –0.70 to –0.33, *P*<.001; I^2^=48%). There was no evidence for an association between duration of treatment and the effect of treatments on depressive symptomatology (χ^2^_1_=0.4, *P*=.50; I^2^=0%).

### Provision of Face-to-Face Contact

As statistical heterogeneity was found in our analysis of treatment effectiveness and in order to exclude the possibility of heterogeneity due to the provision of face-to-face contact, trials that incorporated face-to-face support were compared to interventions that did not use live contact with a therapist. We hypothesized interventions that included face-to-face sessions would show greater reductions in depressive symptoms than treatments that did not. Contrary to what was expected, effect size in treatments that did not use face-to-face support was larger (SMD –0.65, 95% CI –0.79 to –0.50, *P*<.001; I^2^=78%) than in interventions that offered live support (SMD –0.28, 95% CI –0.52 to –0.03, *P*=.03; I^2^=50%). An association between the effectiveness of treatments and the provision of face-to-face contact was found (χ^2^_1_=6.34, *P*=.01; I^2^=84%).

### Utilization of Cognitive Behavioral Therapy

We made the hypothesis that technology-based interventions using CBT techniques are more effective than treatments that do not use components of CBT, reflecting on the predominance of CBT in the literature. Effect size varied only slightly between trials that were based on CBT (SMD –0.58, 95%CI –0.72 to –0.45, *P*<.001; I^2^=71%) and interventions that did not use a CBT protocol (SMD –0.56, 95% CI –0.96 to –0.16, *P*=.006; I^2^=85%). Also, according to the test for subgroup differences, there was no association between the effect of technology-supported treatments on depressive symptoms and utilization of CBT methods (χ^2^_1_=0, *P*=.90; I^2^=0%).

### Severity of Baseline Depression

Comparing trials in which patients showed higher depression scores (BDI ≥25 or PHQ ≥15) with trials in which patients had lower scores (BDI <25 or PHQ <15), technology-based treatment showed to be effective in both groups (higher severity: SMD –0.61, 95% CI –0.79 to –0.44, *P*<.001; I^2^=77%; lower severity: SMD –0.54, 95% CI 0.75 to –0.33, *P*<.001; I^2^=75%). The test for subgroup differences resulted in a chi-square value of 0.3 (df=1, *P*=.59; I^2^=0%).

### Age of Participants

Age did not show a significant impact on effectiveness of technology-based interventions for the treatment of depression. Although effect sizes varied between adults (SMD –0.63, 95% CI –0.79 to –0.46, *P*<.001; I^2^=79%), adults excluding older adults (SMD–0.48, 95% CI –0.69 to –0.27, *P*<.001; I^2^=55%), older adults (SMD –0.53, 95% CI –1.52 to 0.46, *P*=.29; I^2^=84%), and adolescents (SMD –0.28, 95% CI –1.45 to 0.88, *P*=.63; I^2^=91%), subgroup analyses pointed in the same direction (see Table 1 for a description of the different age categories). The effect of technology-based interventions on symptoms of depression was not associated with patient age (χ^2^_3_=1.4, *P*=.71; I^2^=0%).

### Type of Control Condition

Comparing technology-based treatments to TAU resulted in a moderate effect (SMD –0.48, 95% CI –0.78 to –0.18, *P*=.002; I^2^=62%) and the comparison of technology-supported interventions for depression to WLC showed a large effect (SMD –0.79, 95% CI –0.93 to –0.64, *P*<.001; I^2^=51%). The test for subgroup differences resulted in a chi-square value of 3.3 (df=1, *P*=.07; I^2^=69.3%).

## Discussion

This systematic literature review had the following objectives: (1) to collect all relevant clinical studies of technology-based interventions that analyzed the effectiveness for the treatment of depression in order to accurately depict the body of literature and (2) to identify a set of system components of technology- and Internet-based interventions for depression. In general, the results are in line with previous analyses and showed that technology-supported interventions, in fact, reduce depressive symptoms [5,6,121]. This study is one of the first that provides an overview of technical components used in the current set of RCT trials that made use of computerized and online interventions on the treatment of depression.

### Principal Results

Forty-five publications with a total number of 7326 randomized and 4519 analyzable participants were included in this systematic review, and most of the interventions analyzed were able to reduce symptoms of depression. The majority of included studies were of fair (60%) to good (33%) quality, and almost every study included analyzed an intervention that was modular in setup and typically lasted for approximately 10 weeks. Thirty-six studies (80%) deployed a CBT approach. This extends the systematic review by Saddichha et al [8], which consisted of 29 RCT studies using CBT. Usually, the programs were aimed to be used about once a week. This is in line with traditional CBT, which is seen as a step-by-step, short-term treatment with weekly or biweekly therapist sessions for 10 to 20 weeks [17].

Subgroup analysis showed that study quality, treatment duration, provision of face-to-face contact, utilization of CBT, and age of participants had relatively small impact on the outcome of the interventions. For study quality, it is plausible because the intervention quality is not inevitably reflected by the study quality. Likewise, there are reasonable explanations that the age of the participants and treatment durations did not have a decisive impact on treatment outcome. We probably overestimated the influence of technology literacy in older participants because our results confirm findings of literature showing comparable treatment results for all age groups. The small difference in effect for treatment durations can also be explained taking into account that traditional therapy has a similar range of time spans to deliver the same amount of structured information and is chosen depending on, for example, severity of illness and level of support [122]. We were surprised that provision of face-to-face therapy did not show significant effects on the treatment outcome. In fact, this confirmed a recent study comparing traditional therapy with a computerized intervention [65].

Interestingly, the use of CBT components also did not show significant effects on treatment outcome compared to interventions that were not based on CBT. Although there exists evidence that other types of intervention can be equally effective, predominance of CBT in traditional therapy as well as in technology-mediated MHIS urged us to test its superiority in our analysis.

As explained earlier, reminders play a decisive role in the engagement of users in mental health interventions. In this systematic review, approximately 45% of all included studies reported to use email reminders and 10 studies (22%) reported providing phone reminders, mostly as an alternative to reminders sent by email. In the included trials, reminders were typically intended to increase motivation and adherence to therapeutic interventions.

With respect to the design and effective use of reminders, research indicates that a variety of factors show an impact on the efficiency of these alerts. Firstly, it was found that there is a high risk that motivational emails provided within a workplace setting are easily ignored as a consequence of a full email inbox [123,124]. Thus, it might be of importance to offer the possibility to choose between mobile phone or email reminders and to customize time points at which users will be reminded to complete program modules. Secondly, regarding the content of expert-initiated contact, it is postulated that contacts delivering behavior-change techniques might be of greater effectiveness than simple messages that prompt users to access the intervention [10]. Thirdly, it is suggested that reminders containing short motivational messages, quotes, or facts might counteract negative feedback cycles that maintain perceptions of low self-worth and associated depressive symptomatology [11,125]. Lastly, it could also be shown that the personalization of reminders and sending them out frequently enhances the effectiveness of treatment [98]. Nevertheless, nonspecific factors, such as encouragement, empathy, and hopefulness of improvement, may also independently enhance therapeutic gains. To conclude, it seems evident that creating a sense of being continuously supported and encouraged is crucial for user engagement, treatment adherence, and buffering against the development of negative feedback cycles. The regular and consistent receipt of well-designed reminders, motivational messages, and tips may thus be a very powerful means of reminding patients that they are actively working on gaining control over their symptoms [11].

Most of the RCTs included made use of the Internet for delivering mental health interventions for depression or depression comorbid with anxiety. The majority of RCTs included in this systematic review (93%, 42/45) did not make use of mobile phones or tablets, which is surprising because the benefits with respect to user engagement and adherence seem apparent. Typically, the interventions required the interaction with the system and many also included the interaction with a therapist (face-to-face or online) and/or peers on the Web. In all, 80% (36/45) of included programs were based on CBT or used elements of CBT. Given the fact that therapeutic interventions for depression are commonly based on CBT techniques and psychoeducation, which follow a stepwise approach and are usually delivered in person by a therapist, these findings support the authors’ premise. Twenty-seven of 45 included studies used tailored content, tailored feedback, and/or tailored reminders. In the opinion of researchers, the adaptation of information to factors that are relevant to one individual or a group of individuals is an important feature in effective health communication [93,126,127]. In fact, van Genugten et al [128] found that interventions that are more flexible in use (easy to handle for both advanced as well as novice users), provide structure (which is comprehensible to the user and that the user knows at what point he or she is in the process), and use default settings are more likely to be effective. With respect to treatment exposure, it is suggested that personally tailored feedback and goal setting are among the important factors related to the use and exposure to Web-based interventions. In the study by Brouwer et al [129], exposure was regarded as the time spent on the website, page views, and the number of times the user logged on. Although there is a relationship between exposure, adherence, and treatment outcomes, focusing on exposure only gives a limited insight into the pattern of usage and adherence [97].

Doherty et al [130] showed that user engagement also depends on depression severity and that users with minimal symptoms engage much less than other groups. To serve the needs of this patient group, users might benefit from more flexibility (eg, in the elements of the intervention they wish to focus on). Therefore, new approaches such as tailoring of interventions that are more lightweight are needed. However, due to the severity of the disorder, patients with more pronounced symptoms face specific difficulties in engaging with the program. Because these patients require more intensive support than patients with less severe symptoms, they should also be given the choice to change the means of support throughout the treatment. Thus, mechanisms are needed that allow for requesting or being offered face-to-face contact even if the user has initially commenced online treatment. We assume that customization of programs is necessary to enhance long-term adherence and outcomes of interventions.

Only a small number of included studies made use of self-monitoring components, such as symptom tracking and tracking reminders, yet they are seen as key features of psychotherapy [11,96] and in behavior-change interventions in general [99,115-118].

Although social support is widely recognized as an important feature in behavior change [119,120], in this analysis only seven studies (16%) used peer support. In the included studies, social support consisted of the use of Web-based discussion boards (asynchronous social support), which intended to provide the ability to connect with other patients using the same intervention. However, the literature shows that there is disagreement with regard to use and benefit of discussion forums and chat rooms. Although many studies support the use of these components [123,131,132], others do not [133-135]. The effectiveness of peer support depends on individual factors, such as perception of the credibility of Internet-based peer advice and perceived quality of interaction [10]. Furthermore, effectiveness might also rely on user involvement. It could be shown that users that actively post and respond to messages are more likely to benefit than users that participate only passively [132].

Regarding visual attractiveness, 44% of all included studies analyzed an intervention that included visually appealing content, such as graphs, illustrations, comics, or photos, which often serve a motivational purpose. Only 11 studies (24%) described the utilization of audio and/or voiceovers. Video footage, often containing case-enhanced learning, was also found in 11 RCTs (24%).

In addition, this analysis showed that game elements were only used in three RCTs and if gaming was included, it was in the form of knowledge quizzes. Relatively few studies have incorporated games as part of their persuasive design.

Although virtual reality has shown to be effective in the treatment of anxiety and pediatric disorders [136], so far there is no study utilizing this technology for the treatment of depression and this might open a promising direction [137]. With respect to virtual agents and synthesized speech, Morrison et al [10] were also not able to show an association between digitized speech and improved outcomes in depression. To date, technological advances and improved design of animations and avatar-based systems are likely to permit the development of sufficiently sophisticated services to simulate real interaction [10] and, therefore, might show more impact on treatment outcomes. Future research is needed that concerns the effectiveness of serious gaming and virtual reality in technology-supported mental health interventions currently underrepresented in literature.

### Limitations and Future Directions

The list of factors that influence user friendliness as well as the different platforms for delivery included in this analysis is not exhaustive. In fact, the majority of RCTs included in this systematic review did not make use of mobile phones or tablets. It is expected that especially newer studies could use different channels of service delivery (eg, mobile phone or tablet delivery). Consequently, studying future interventions that make use of these delivery channels would give an interesting insight into the influence of different modes of delivery on treatment effectiveness.

Further limitations are related to the strict process of study selection applied in this systematic review. Many trials were excluded because (1) they were not described as being randomized, (2) participants showed no symptoms of depression at baseline, (3) they included other mental health disorders, and (4) they did not assess one of the outcomes of interest. In fact, the decision to only include RCTs might lead to potential limitations of this systematic review. Even though RCTs are regarded as the “gold standard” of reliable evidence, the criteria to only include RCTs might lead to the exclusion of relevant articles that examined the effectiveness of MHIS, but used a different study design. Primarily, the exclusion of non-RCTs in this review lead to a facilitated analysis of studies because differences in methodological quality are, although not completely removed, limited. A possible consequence of this decision might be that we missed studies of newer interventions, which might not yet be evaluated in an RCT study format because they are undergoing their piloting phase at the current time [15,104].

In addition, as a consequence of considering a wide range of MHIS, included trials differed considerably in the type of therapeutic programs they used, baseline depression severity, age of participants, duration of treatment, type of control condition, methodological quality, and the various system components they utilized to enhance user engagement, motivation, and effectiveness of the intervention. As a consequence of this moderate-to-high level of heterogeneity across included trials, comparability is restricted and results should be handled with care. Subgroup analyses demonstrated that there is, in fact, a significant association between the effectiveness of interventions and the provision of face-to-face contact as well as the type of control they used. As previously noted, the inclusion criteria stated in the protocol also included other mental disorders such as anxiety disorders. However, the vast number of articles identified in the electronic search posed an additional challenge. Consequently, it was decided to focus on depression and depression comorbid with anxiety only. Considering that many mental health problems often co-occur [138], study findings might be constrained as a result of this relatively strict inclusion/exclusion of certain mental disorders.

Because technology-based psychological interventions adapt established methods of treatment and only the means of delivery are altered, the issue of noninferiority plays a major role in this review. With respect to the overall effectiveness of technology-based interventions, it is of utmost importance to review the literature from the perspective of noninferiority trials that compare an established evidence-based treatment (eg, CBT) with a new one (eg, technology-based CBT). It also needs to be clarified that the absence of a significant difference between two interventions in an RCT cannot be equated with noninferiority and that the comparison of treatment effects between studies are only appropriate if the new and existing treatments are compared against a reference that does not substantially differ in methods and population [2].

In addition, identifying the points of disengagement and gaining deeper insight into the patterns of program usage is crucial for the refinement of system components that are most strongly associated with user engagement and symptom improvement. Data on patterns of use further offer an opportunity to refine content, means of delivery and to adapt both to the needs and preferences of specific groups of users [130]. Additional research is needed to overcome these shortcomings by assessing the association of patterns of component use and the improvement of symptoms by means of advanced statistical analyses. Furthermore, it is suggested that RCTs assess symptom reduction more frequently to obtain improved information about nonlinear relationships between patterns of usage and therapeutic gains. To enhance the understanding of when and how to choose and use different system components, it also is important to comprehend the dose-effect relationship of different components [128]. Future studies should be aimed to clarify the causal relationship between patterns of program usage and symptom improvement by assigning participants to different system components. In addition, it is likely that other predictors such as age, gender, and education affect the relationship between usage of system components, engagement, and outcomes. Therefore, additional studies are needed to assess whether differences exist between those populations.

In regard to designing mental health interventions, it is of utmost importance to understand what users need and expect from computerized or Web-based mental health interventions and how individuals rate different system components with respect to usefulness, practicality, connectivity, time demands, professional support, social interaction, convenience, novelty, reliability, confidentiality, trustworthiness, motivation, and engagement. Qualitative feedback offers a solution to find answers to the proposed questions. Likewise, user feedback might disclose disparities between user expectations and actual results. It can be assumed that user preferences vary greatly from individual to individual. This, in turn, supports the rationale of customization and tailoring of programs to create unique user experiences based on client’s preferences without losing the effectiveness of interventions.

Moreover, to clarify the clinical feasibility of computer- and Web-supported mental health interventions, it also is important and worthwhile to repeatedly listen to the opinion of therapists. Apart from the time and cost savings, there is a need for a thorough understanding of the program, which could be achieved by the provision of a protocol, printed manual, and an overview of the program to the therapists involved. In addition, more detailed information on their practices and how to deal with clients who are not engaging with the program should be provided [130].

Although clinicians tend to be very self-protective about their time commitments and skeptical about technology [139], value in health care should always be defined around the customer. Nonetheless, because value in health care is measured by the outcomes achieved, value depends on results not input [140]. First, computer-supported interventions should be designed and modified to optimize clients’ benefits. Although value in health care is measured by the outcomes achieved, only assessing the effect on symptom reduction fails to include the value of mediators that might explain behavior change (eg, factors such as skills, attitudes, and self-efficacy) [128]. Thus, a further shortcoming of the current meta-analysis is the lack of analyses of potential mediators of the effect.

### Implications and Recommendations

From a system component perspective, there is a strong need to counteract the decrease of program usage over the course of the intervention that is typically found in unguided technology-mediated interventions for mental health [118,141,142]. Thus, it is of great importance to implement the program components that are associated with improved and regular program engagement. One of the factors that have been linked to a decrease in module completion rate is obliging users to complete module sessions in a predetermined sequence [143]. On the one hand, the delivery of module sessions in a tunneled format bears many advantages, such as a greater number of website pages accessed, greater time spent on the website, and greater knowledge gained from the website [143]. On the other hand, tunneling might lead to a decrease in module completion because users are required to complete the between-session homework assignments before being able to start the next module session [11]. Therefore, developers have to enable a high level of flexibility in the choice of relevant modules as well as the speed with which users proceed through the modules to keep users engaged. One approach to allow for a greater flexibility, if not violating the underlying psychological theory, is to make homework assignments optional [11] or to leave it to the user at what stage they prefer to complete the homework tasks. Future studies are needed to confirm whether the provision of more user-driven programs might offer an advantage over the frequently used linear delivery of interventions in mental health interventions [139].

In addition, little is known about the synergistic effects of behavior-change components, modes of delivery, and user friendliness. Van Genugten et al [128] found synergistic effects in interventions that made use of a specific CBT components in combination with the provision of rewards. In general, little synergistic effects were found. Thus, there is a need to further assess the cumulative effect of different system components on treatment effectiveness and to analyze how specific combinations affect behavior change. To fully determine the most optimal delivery mode, further studies are needed that randomly assign participants to different platforms for delivery [128].

Although no consensus with respect to effectiveness of online peer support was reached yet [94,95], anonymous online support groups and discussion forums might help users to overcome the feeling of being stigmatized by connecting patients with others. A further advantage of these social support components is that time and location are no longer obstacles for active participation [96]. Nevertheless, further research is needed concerning the type of online support, such as expert-led or user-driven, moderated or nonmoderated, and synchronous (eg, chat rooms) or asynchronous (eg, discussion forums) [96]. From a design and engagement perspective, a nuanced view on target groups suggests that complementarity between content of interventions that target different mental disorders is crucial when designing computerized and Web-based mental health interventions. Patients with multiple disorders present a considerable challenge in the design of technology-supported interventions and, as a consequence, are often excluded from studies even though they might profit from certain components. One reason for the exclusion of comorbid and multimorbid patients is the pressure toward relatively stringent and precisely defined interventions, which are amenable to RCTs [130]. Regarding the high comorbidity of mental disorders, it is of greatest importance that this topic is given more attention in the design of technology-amenable interventions.

### Conclusions

The development of MHIS targeting the change in health behavior requires great expertise and a thorough understanding of the problem area, underlying therapeutic strategies, and the design of persuasive systems. The findings of this systematic review contribute to the body of knowledge on the effectiveness of technology-supported therapeutic interventions for the treatment of depressive symptoms. This work is intended to provide a basis for the assessment of the impact of specific system components on treatment effects in RCTs of technology- and Web-based interventions for depression. Thus, the overall goal of this review was to identify such components and to enhance the understanding of the mechanisms through which technology-enabled interventions exert their therapeutic benefits by means of such.

Further quantitative studies are needed to assess the impact of identified components and to identify other system components that are relevant for the design of future technology-mediated MHIS for the treatment of depression and other mental disorders.

Because of the high relevance of the anatomy of MHIS, attention should be paid to design issues when developing new eHealth services in the future. To enhance dissemination and utilization of technology-based MHIS, the focus needs to be not only on how the interventions affect users, but also on how patients use and interact with technology and one another through them. Therefore, future studies are needed that add to the body of knowledge of technology-supported interventions for the treatment of depression by assessing patterns of program usage and user engagement.

To conclude, health information technology is a fast-growing field of research, which has the potential to effectively treat people suffering from mental disorders. Despite that, there is still room for improvement in the design of technology-based interventions for the treatment of depression. The delivery of interventions via technology is a promising and cost-effective approach to diminish the significant treatment gap and the various barriers associated with the disorder.

## Appendix

Multimedia Appendix 1 Subgroup analyses by study quality. Q>3.5: high quality studies; Q<= 3.5: low quality studies.

Multimedia Appendix 2 Subgroup analyses by duration of treatment. Comparison of studies with a duration of equal or less than 10 weeks with trials of more than 10 weeks duration.

Multimedia Appendix 3 Subgroup analyses by face-to-face contact. Comparison of studies that made use of face-to-face therapy (F2F_Y) with those that did not use live therapist contact (F2F_N).

Multimedia Appendix 4 Subgroup analyses by the use of a CBT protocol. Comparison of interventions that were based on CBT (CBT_Y) with treatments that did not make use of CBT techniques (CBT_N).

Multimedia Appendix 5 Subgroup analyses by severity of baseline depression. Comparison of interventions that studied patients with a high level of baseline depression (SEV_H) with trials that included patients with a low level of baseline severity (SEV_L).

Multimedia Appendix 6 Subgroup analyses by age of included participants. Comparison of trials in A) adult patients (AGE_1) with B) trials that studied effects in adults excluding older adults (AGE_2), C) trials in older adults only (AGE_3), and D) trials that studied the effect on symptoms of depression in adolescents only (AGE_4).

Multimedia Appendix 7 Subgroup analyses by type of comparator. Comparison of trials with a control group that received treatment as usual (TAU) with RCTs that compared the intervention to a waiting list control (WLC) group.

## Abbreviations

BDI: Beck Depression Inventory
CBT: cognitive behavior therapy
HIS: health information system
MHIS: mental health information system
PHQ: Patient Health Questionnaire
RCT: randomized controlled trial
SMD: standard mean difference
SMS: short message service
TAU: treatment as usual
WLC: waiting-list control
