# Supplementary figures and images for: Toward the Design of Evidence-Based Mental Health Information Systems for People With Depression: A Systematic Literature Review and Meta-Analysis
Source: J Med Internet Res. 2017 May 31;19(5):e191. doi: 10.2196/jmir.7381 (PMC5471345; doi:10.2196/jmir.7381)

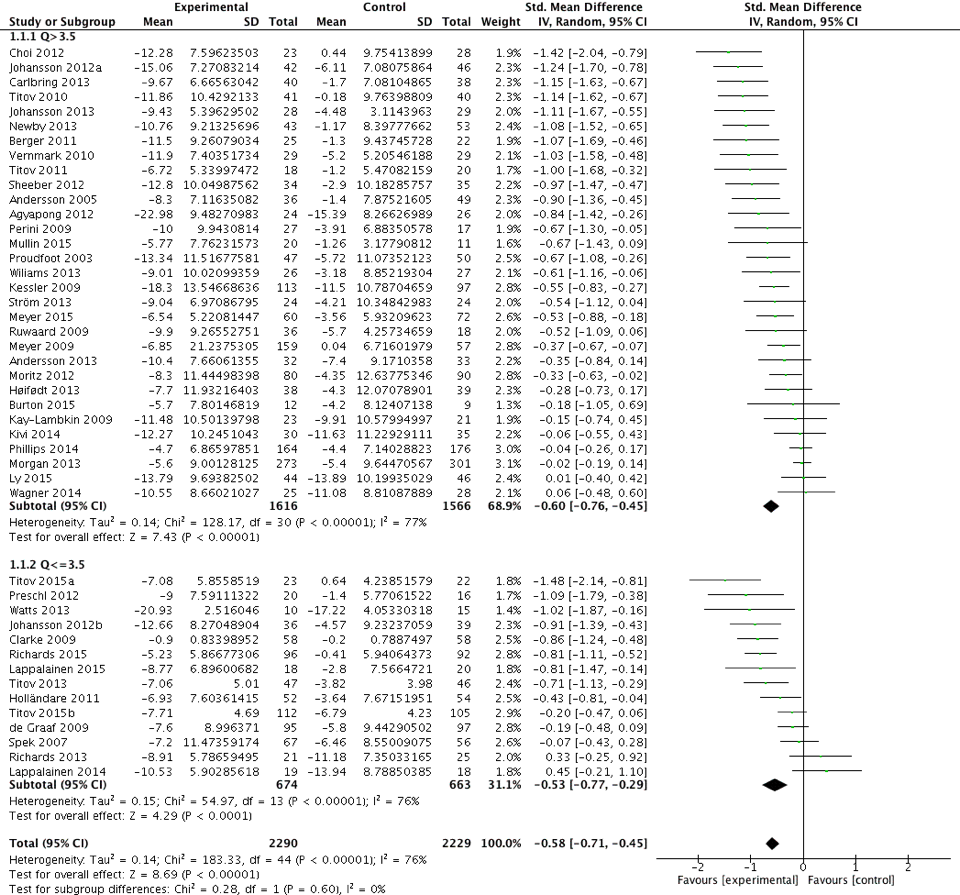

Supplement: Multimedia Appendix 1 [file jmir_v19i5e191_app1.png]

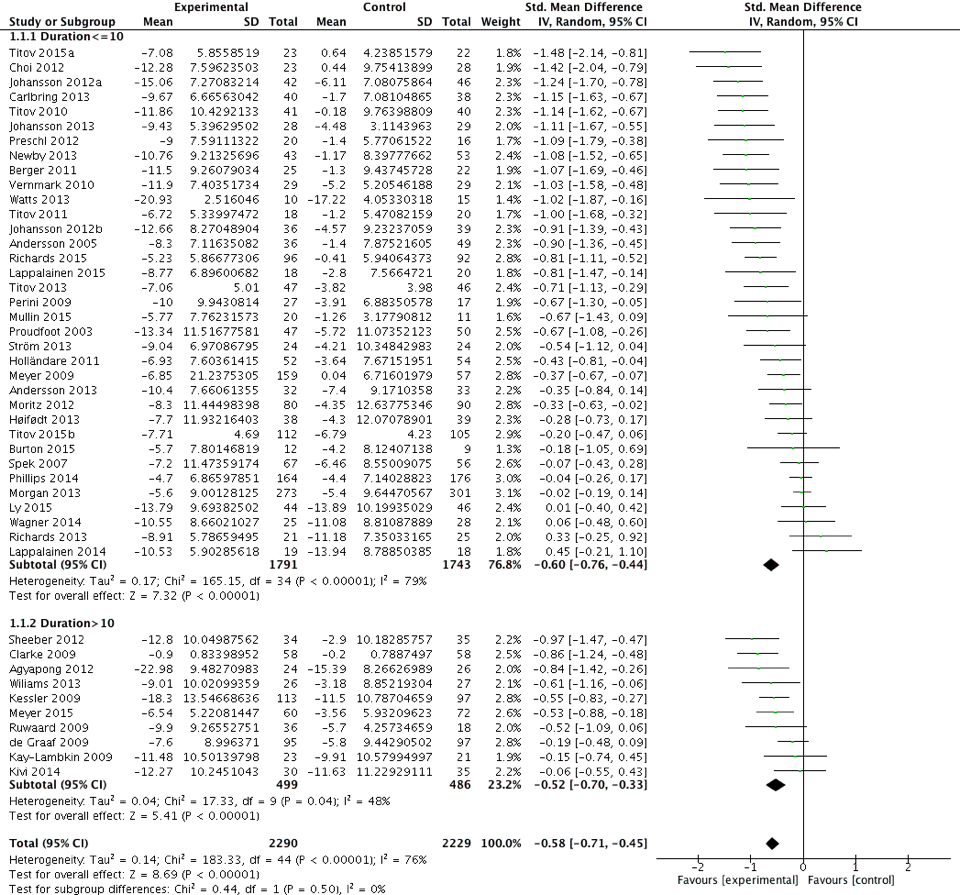

Supplement: Multimedia Appendix 2 [file jmir_v19i5e191_app2.png]

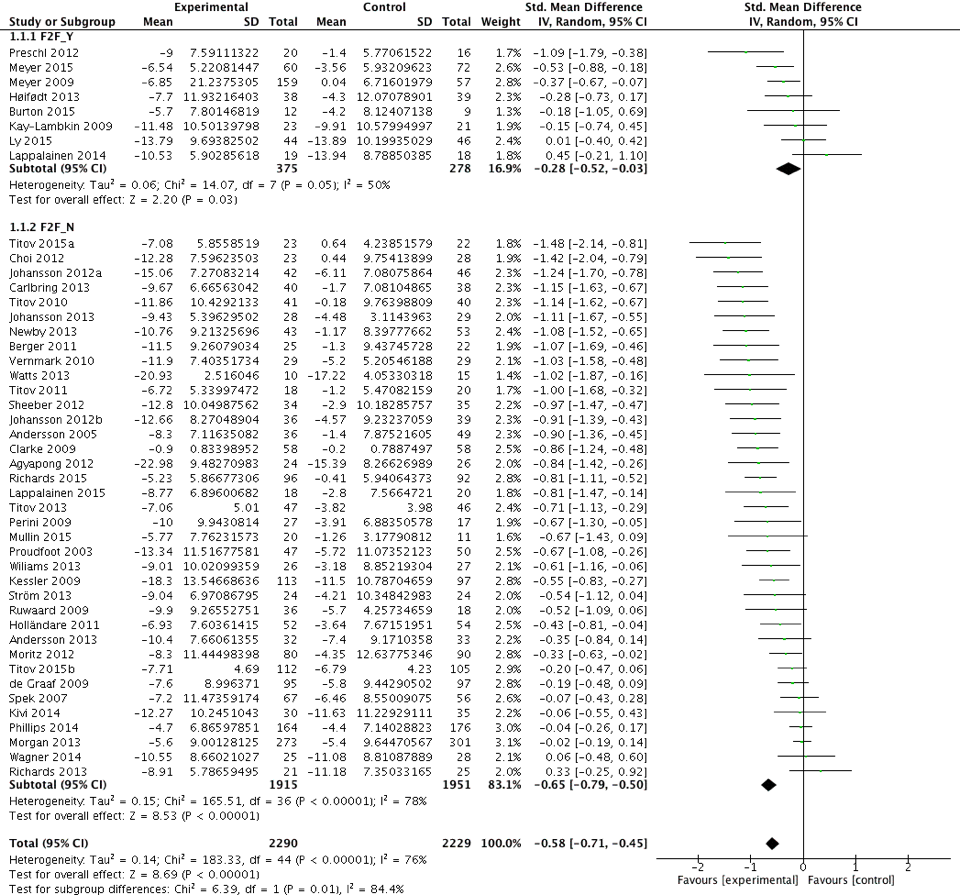

Supplement: Multimedia Appendix 3 [file jmir_v19i5e191_app3.png]

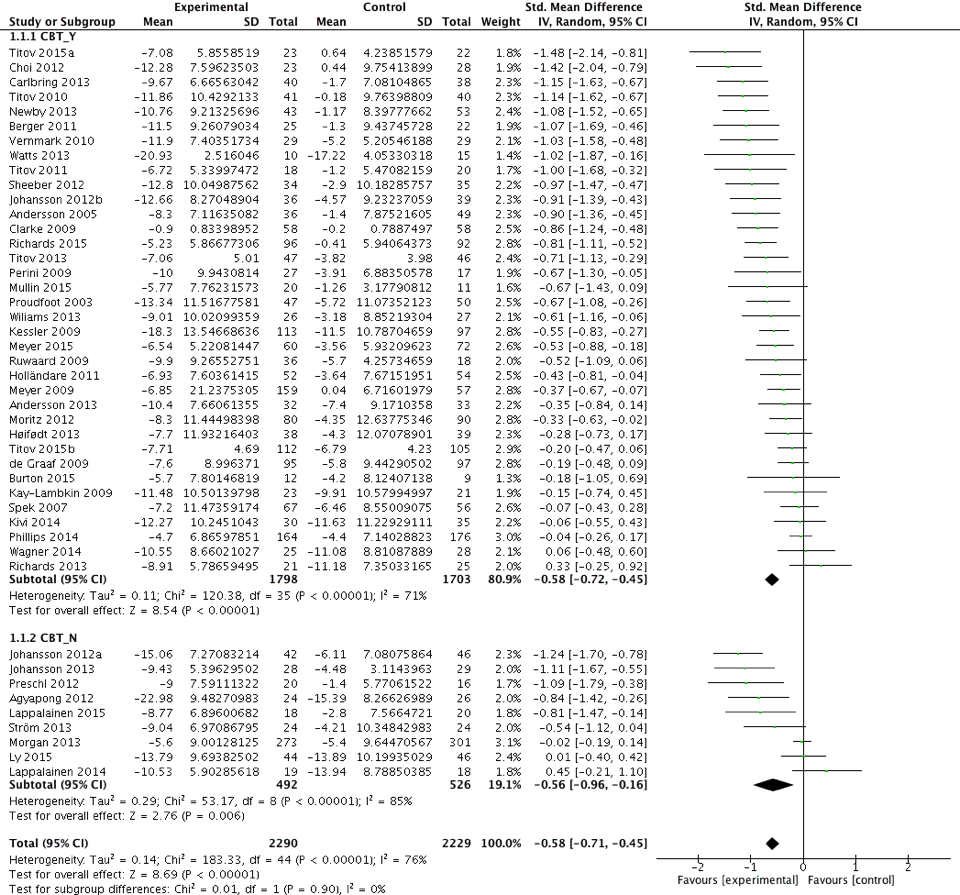

Supplement: Multimedia Appendix 4 [file jmir_v19i5e191_app4.png]

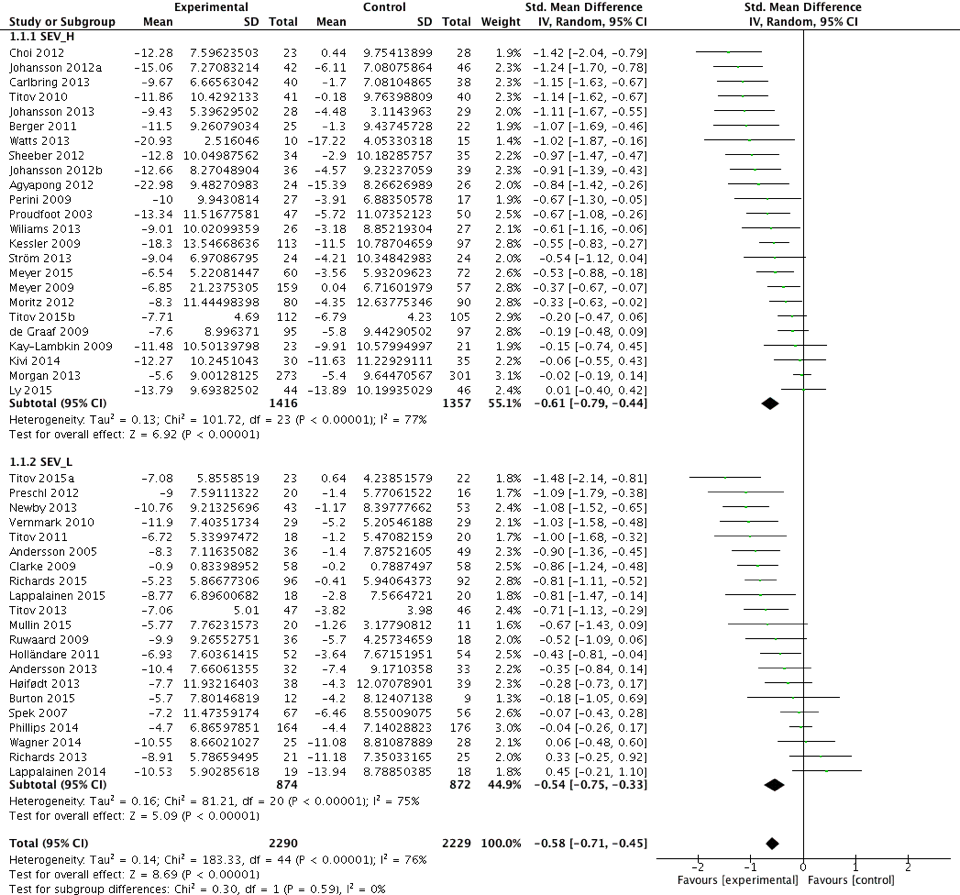

Supplement: Multimedia Appendix 5 [file jmir_v19i5e191_app5.png]

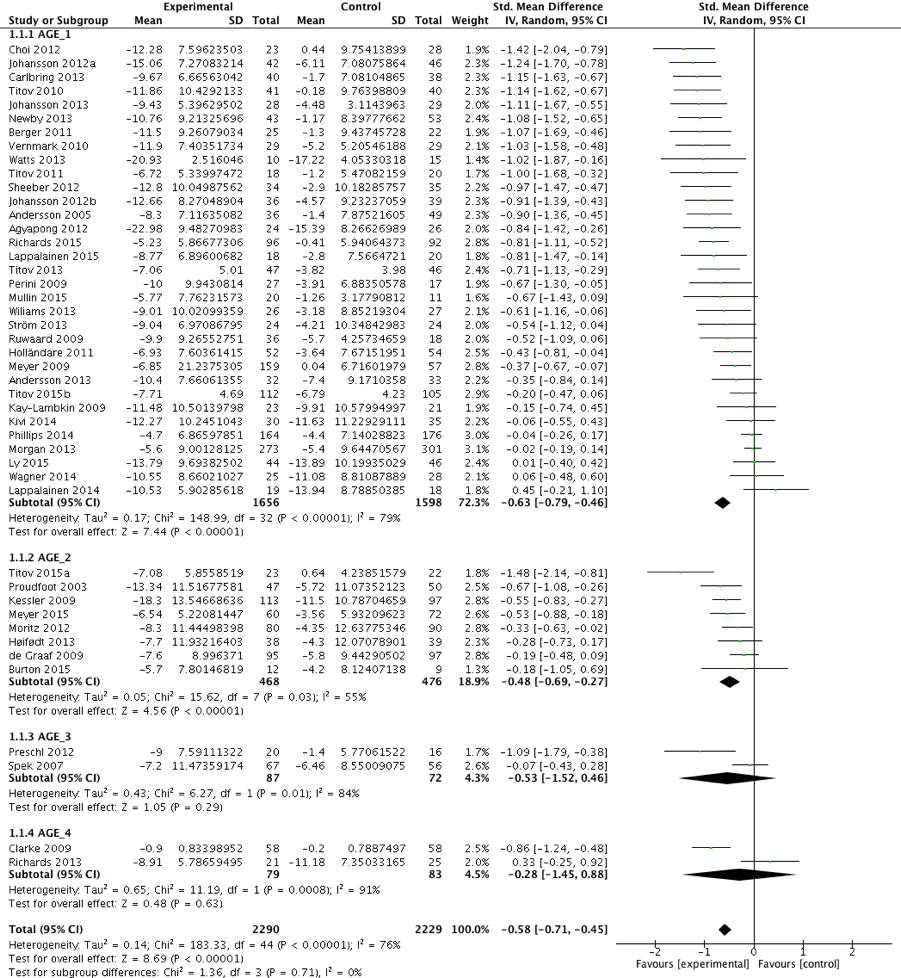

Supplement: Multimedia Appendix 6 [file jmir_v19i5e191_app6.png]

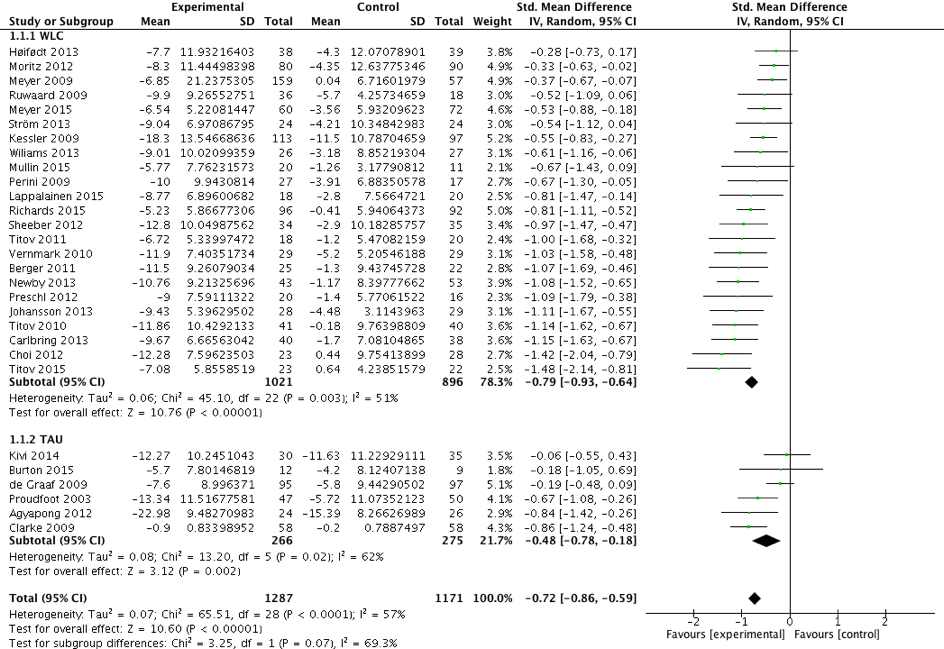

Supplement: Multimedia Appendix 7 [file jmir_v19i5e191_app7.png]
